# Individual and joint estimation of humpback whale migratory patterns and their environmental drivers in the Southwest Atlantic Ocean

**DOI:** 10.1038/s41598-022-11536-7

**Published:** 2022-05-06

**Authors:** Luis Bedriñana-Romano, Alexandre N. Zerbini, Artur Andriolo, Daniel Danilewicz, Federico Sucunza

**Affiliations:** 1 Instituto de Ciencias Marinas y Limnológicas, Facultad de Ciencias, Universidad Austral de Chile, Casilla 567, Valdivia, Chile; 2 NGO Centro Ballena Azul, Valdivia, Chile; 3 Centro de Investigación Oceanográfica COPAS Coastal, Universidad de Concepción, Región del Bio Bio, 4070043 Concepción, Chile; 4 Cooperative Institute for Climate, Ocean and Ecosystem Studies, University of Washington and Marine Mammal Laboratory Alaska Fisheries Science Center/NOAA, 7600 Sand Point Way NE, Seattle, WA USA; 5 Marine Ecology and Telemetry Research, 2468 Camp McKenzie Tr NW, Seabeck, WA 98380 USA; 6 Instituto Aqualie, Av. Dr. Paulo Japiassú Coelho, 714, Sala 206, Juiz de Fora, MG 36033-310 Brazil; 7 Laboratório de Ecologia Comportamental e Bioacústica, LABEC, Departamento de Zoologia, Instituto de Ciências Biológicas, Universidade Federal de Juiz de Fora, Juiz de Fora, Minas Gerais Brazil; 8 Grupo de Estudos de Mamíferos Aquáticos do Rio Grande do Sul (GEMARS), Porto Alegre, RS Brazil

**Keywords:** Animal migration, Ecological modelling, Ecology, Marine mammals

## Abstract

Humpback whales (*Megaptera novaeangliae*) perform seasonal migrations from high latitude feeding grounds to low latitude breeding and calving grounds. Feeding grounds at polar regions are currently experiencing major ecosystem modifications, therefore, quantitatively assessing species responses to habitat characteristics is crucial for understanding how whales might respond to such modifications. We analyzed satellite telemetry data from 22 individual humpback whales in the Southwest Atlantic Ocean (SWA). Tagging effort was divided in two periods, 2003–2012 and 2016–2019. Correlations between whale’s movement parameters and environmental variables were used as proxy for inferring behavioral responses to environmental variation. Two versions of a covariate-driven continuous-time correlated random-walk state-space model, were fitted to the data: i) Population-level models (P-models), which assess correlation parameters pooling data across all individuals or groups, and ii) individual-level models (I-models), fitted independently for each tagged whale. Area of Restricted Search behavior (slower and less directionally persistent movement, ARS) was concentrated at cold waters south of the Polar Front (~ 50°S). The best model showed that ARS was expected to occur in coastal areas and over ridges and seamounts. Ice coverage during August of each year was a consistent predictor of ARS across models. Wind stress curl and sea surface temperature anomalies were also correlated with movement parameters but elicited larger inter-individual variation. I-models were consistent with P-models’ predictions for the case of females accompanied by calves (mothers), while males and those of undetermined sex (males +) presented more variability as a group. Spatial predictions of humpback whale behavioral responses showed that feeding grounds for this population are concentrated in the complex system of islands, ridges, and rises of the Scotia Sea and the northern Weddell Ridge. More southernly incursions were observed in recent years, suggesting a potential response to increased temperature and large ice coverage reduction observed in the late 2010s. Although, small sample size and differences in tracking duration precluded appropriately testing predictions for such a distributional shift, our modelling framework showed the efficiency of borrowing statistical strength during data pooling, while pinpointing where more complexity should be added in the future as additional data become available.

## Introduction

Humpback whales (*Megaptera novaeangliae*) present a migratory scheme characterized by a segregation between summer feeding grounds at higher latitudes and winter breeding/calving grounds at lower latitudes where feeding events are rare^1–3^. In general, populations present a high degree of philopatry in wintering and summering grounds, defined migratory corridors and clear trends on migration timings^4–9^. Nevertheless, behavioral variation from this general scheme has been described in terms of alternative migratory routes, feeding events at mid-latitudes, overwintering at higher latitudes, delay on migration onset and sex-segregated migratory patterns^1,10–15^. One of the factors controlling this variation has been hypothesized to be interannual shifts in foraging success at feeding grounds, mainly for mature females^16^. Feeding grounds at polar regions are currently experiencing major ecosystem modifications^17–19^, therefore quantitatively assessing behavioral responses to habitat characteristics^20–22^ and physiological repercussions^23,24^ are crucial for understanding population-level outcomes derived from such modifications^25,26^.

Movement models fit to telemetry data can be used for estimating animal behavioral shifts and how they relate to environmental covariates^27–29^. Moreover, analytical assessments of movement patterns might be accommodated for addressing estimation of movement parameters across individuals, which might be considered a tool for scaling up individual estimates to the population-level^30,31^. Although, joint estimation of these movement parameters provides an efficient approach, how inter-individual variation is handled is crucial for the inference process. Complete polling across individuals (*i.e.* all individuals provide information for a single set of movement parameters) allows the highest levels of statistical strength borrowing^30^. However, if large variation exists at this level (*i.e.,* different groups such as adults and juveniles or males and females, show distinct movement patterns) complete polling might yield average parameter estimates that are not representative of the underlaying biological process. Hence, assessing both individual and population variability in movement characteristics is desirable.

Our goal here was to implement this double approach (individual and joint estimates) to the case of a humpback whale population in the Southwest Atlantic Ocean (SWA). This population was heavily depleted by whaling operations during the 19th and early twentieth centuries, but has since then been experiencing nearly complete recovery^32–34^. SWA humpback whales migratory connections between their neritic breeding habitats off the central and northeast coast of Brazil^35,36^ to feeding destinations in high latitudes near the Scotia Sea, have been reported^37–42^. However, their migratory behavior after departing the Brazilian coast and the environmental factors that influence their preference for certain areas at the feeding grounds are poorly understood. Tracking duration after departing Brazilian waters might pose a critical data limitation for inferring selection patterns at the feeding grounds. Henceforth, in this case models that jointly estimates parameters regulating movement responses to environmental variables might optimize data usage. Comparisons between the complete-pooling approach (including grouping by sex or tagging period), and individual level assessments were made to explore the required level of model complexity for this population.

## Methods

### Tagging and telemetry data

Tagging procedures were described in detail in previous studies^7,42,43^. Briefly, tagging efforts have been conducted in the breeding grounds off Abrolhos Bank and off the northern coast of Bahia State, north of Abrolhos, Brazil, during the austral spring of two periods, the first considering 2003- 2012 and a second period from 2016 to 2019. Tags were deployed from small boats using a custom-modified compressed-air line-throwers ARTS/RN, Restech Norway^44^, set at pressures ranging between 10 and 15 or a custom-made 8 m long fiberglass pole^45^ at about 4 to 5 m from the whale. Multiple types of custom-designed fully implantable satellite tags were used^20,46,47^ configured as location-only (models SPOT3, SPOT5 and SPOT6 [2003–2012]) or archival (MK/SPLASH10 [2016–2019]) tags manufactured by Wildlife Computers (Redmond, Washington, USA).

As we were concerned with understanding the environmental drivers associated with the initialization of foraging related behavior at potential feeding grounds, some data caveats needed to be addressed. By using relocation data only, foraging behavior cannot be determined. As a proxy for this, movement characteristics such as low velocity and low directional persistence (Area of Restricted Search, ARS) have been proposed^27,28^. Data from breeding grounds in Brazil and potential feeding grounds in the Southern Ocean are likely to elicit such ARS characteristics, which might include extremely dissimilar, yet analytically indistinguishable, behaviors (negligible feeding occurs at breeding grounds in humpback whales^48^). To overcome this limitation, we removed locations at breeding grounds, before the onset of a clear sign of migratory behavior, using the continental shelf off Brazil as cutting point. Transmission loss during migration posed another challenge as many whales never reached areas where ARS behavior would be predominant. Therefore, data from individuals that ceased transmission before reaching the Polar Front (~ 50°S) were also discarded. This limit was based on previous observations of SWA humpback whales reducing their speed around the average location of the Polar Front^42^, here considered a geographical boundary from where ARS behavior was likely to be initialized after migration. This allowed us to reasonably compare speed variation among groups of whales between migratory and foraging areas, as well as to estimate migratory duration. After data censoring, location data from the reminder 22 individual whales (Table 1) were filtered using the R package *argosfilter*^49^ for removing obvious extreme positions (those implying velocities larger than 5 m s^-1^).

**Table 1:** Summary of satellite tag deployments and tracking data for 22 humpback whales used during analyses.

ID	Sex	CP	SR	Locs	Start	End	Date50s	M_time	TD	ICE08%	ICE10%
2003-24,642	F	Yes	Mo	504	2003-12-28	2004-05-18	2004-02-02	36	142	59.1	61.5
2005-10,946	F	Yes	Mo	132	2005-10-25	2006-01-03	2005-12-02	38	70	17.4	34.1
2005-24,641	F	Yes	Mo	159	2005-10-27	2006-02-08	2005-12-26	60	104	10.7	11.3
2009-87,783	F	Yes	Mo	577	2009-10-19	2010-02-03	2009-12-29	71	107	21.8	0.0
2009-87,771	U	No	Ad	362	2009-09-21	2009-11-13	2009-10-19	28	53	35.6	13.5
2012-111,871	F	Yes	Mo	2385	2012-11-25	2013-04-20	2013-01-02	38	146	52.8	73.9
2012-121,189	M	No	Ad	1546	2012-10-25	2013-07-26	2012-12-14	50	274	61.3	61.1
2012-87,632*	F	Yes	Mo	1335	2013-01-20	2013-04-13	NA	NA	83	100.0	100.0
2017-172,000	U	No	Ad	781	2017-12-07	2018-01-21	2018-01-03	27	45	0.0	0.0
2017-172,002	F	Yes	Mo	1763	2017-11-16	2018-03-11	2017-12-21	35	115	7.1	5.0
2017-84,484	M	No	Es	704	2017-12-05	2018-01-14	2018-01-07	33	40	0.0	0.0
2017-111,870	M	No	Un	1849	2017-11-03	2018-02-07	2017-12-01	28	96	0.0	0.0
2017-172,001	F	Yes	Mo	2226	2017-11-28	2018-03-17	2017-12-26	28	109	45.0	29.2
2017–121,203	M	No	Es	2051	2017-10-26	2018-02-05	2017-11-22	27	102	21.2	19.3
2017-120,937	M	No	Es	1733	2017-10-30	2018-01-19	2017-11-24	25	81	0.0	0.0
2018-84,485	F	Yes	Mo	810	2018-12-04	2019-01-24	2019-01-04	31	51	0.0	0.0
2018-112,696	F	Yes	Mo	700	2018-10-26	2018-12-06	2018-11-29	34	41	0.0	0.0
2018-172,008	M	No	Es	2252	2018-10-22	2019-02-06	2018-11-26	35	107	67.2	69.5
2018-121,191	F	Yes	Mo	894	2018-11-29	2019-01-17	2019-01-09	41	49	0.0	0.0
2018-171,994	M	No	Es	1291	2018-10-25	2018-12-22	2018-11-30	36	58	0.0	0.0
2019-194,591	U	No	Ad	1204	2019-10-27	2020-01-10	2019-12-16	50	75	0.0	0.0
2019-194,601	F	Yes	Mo	402	2019-10-24	2019-12-10	2019-12-01	38	47	0.0	0.0
Mean				1166.4				37.6	90.7		
Median				1049				35.0	82.0		
SD				716.6				13.9	52.0		

The first four digits on the identification code (ID) correspond to tagging year. The sex column denotes whether whales were female (F), male (M) or undetermined (U). CP indicates calf presence. SR denotes the social role of each whale, with four possible categories, mother (Mo), adult (Ad), escort (Es) and undetermined (Un). Locs correspond to the number of individual positions available for each whale. Start and End correspond to the date range of tracking duration after removing locations on Brazilian shelf areas (before migration onset). The date each whale crossed 50°S (date50S) and the number of days this migratory displacement lasted (M_time) are also provided. TD correspond to tracking duration expressed in days. ICE08% and ICE10% correspond to the percentage of the data located in areas with sea ice coverage during August and October, respectively. Asterisk denotes a whale that presented a large data gap during migration, henceforth analyzed tracking data is entirely located south of the Polar Front.

### Sex and social role determination

Biopsy sampling was carried out along tagging procedures. Skin samples were collected using crossbows^50^ and sex was identified through molecular methods as described in^42^. The social role of the tagged individuals was defined at the moment of tagging and was based on the position of the animals in the group composition. Testing differences in movement parameters considering all possible combinations between sex and social role categories would have been unfeasible with the limited number of whales considered here (Table 1). Therefore, during modelling only two categories were considered. The first category included only females, which were all adults accompanied by calves (mothers). The second category was more heterogeneous and included males and those of undetermined sex, with different social roles (males +).

### Oceanographic covariates

Chlorophyll-*a* (CHL), sea surface temperature (SST), sea surface temperature anomaly (SSTA), wind stress curl (CURL), sea ice fraction, and depth data were extracted using R package rerddapXtracto^51^, which accesses the ERDDAP server at the NOAA/SWFSC Environmental Research Division. CHL data were retrieved from satellite level-3 images from the Moderate Resolution Imaging Spectroradiometer (MODIS) sensor onboard the Aqua satellite (Dataset ID: erdMH1chlamday), corresponding to monthly averages. For SST, SSTA and sea ice fraction, data were obtained from daily averages of level-4 satellite images derived from the Multi-Scale Ultra-High Resolution (MUR) SST Analysis database (Dataset ID: jplMURSST41 for SST and sea ice fraction; Dataset ID: jplMURSST41anom1day for SSTA). MUR-SST maps merge data from different satellites, combined with in situ measurements, using the Multi-Resolution Variational Analysis statistical interpolation^52^. Daily SSTA are constructed as the difference between daily SST data and the daily mean SST from a climatology ranging from 2003 to 2014. CURL data (Dataset ID: erdlasFnTran6_LonPM180) is calculated from geostrophic winds based on U.S. Navy Fleet Numerical Meteorology and Oceanography Center analyzed fields of sea level pressure and is provided on a 6-hourly basis. Sea ice fraction data are provided daily in a scale from 0 to 1. As most of these data were zeros during whales´ migratory displacements, we generated new binary data, with one indicating the presence of sea ice over a ten-day sample (evenly spaced) during August (ICE08) and October (ICE10) of each year. In addition, we retrieved the average position of the Southern Boundary of the Antarctic Circumpolar Current (SBACC) from the Committee on the Earth Observation Satellites (CEOS). This processed file is made available by the Australian Antarctic Data Centre, through CEOS, based on data from^53^. The distance of each whale location to SBACC (DSB) was also incorporated as a covariate in the models. A summary of covariate data can be found in Table 2.

**Table 2:** Variables used in movement models.

Variable	Abbreviation	Units	Spatial resolution	Temporal resolution	Source
Chlorophyll-a	CHL	mg C m^−3^	4.64 × 4.64 km	Monthly	ERDDAP
Sea surface temperature	SST	°C	0.01 × 0.01 degree	Daily	ERDDAP
Sea surface temperature anomaly	SSTA	°C	0.01 × 0.01 degree	Daily	ERDDAP
Wind stress curl	CURL	MPa m^−1^	1 × 1 degree	Six-hourly	ERDDAP
Presence of ice during August	ICE08	Binary	0.01 × 0.01 degree	Yearly	ERDDAP*
Presence of ice during October	ICE10	Binary	0.01 × 0.01 degree	Yearly	ERDDAP*
Distance to the average position of southern boundary of the Antarctic circumpolar current	DSB	Meters	1 × 1 km	Static	CEOS*
Depth	DEPTH	Meters	0.016 × 0.016 degree	Static	ERDDAP

Asterisks denote data modification after gathering from source (see “Methods”).

### Modeling approach

We fitted a continuous-time correlated-random-walk model (CTCRW) which estimates two state variables, velocity and true locations from error-prone observed locations, and two parameters, *β* controlling directional persistence and *σ* controlling the overall variability in velocity^54^. We used a modified CTCRW that considers *β*_*t*_ and *σ*_*t*_ to be random variables that vary among continuous time intervals *t* (expressed in hours) as a function of environmental covariates^55^.

$${\varvec{log}}\left( {{\varvec{\sigma}}_{{\varvec{t}}} } \right)\sim {\mathbf{Normal}}\left( {{\varvec{\mu}}_{{1,{\varvec{t}}}} ,{\varvec{\varepsilon}}_{1} } \right)$$

$${\varvec{\mu}}_{{1,{\varvec{t}}}} = {\varvec{A}}0 + \user2{A*X}_{{\varvec{t}}}$$

$${\varvec{log}}({\varvec{\beta}}_{{\varvec{t}}} )\sim {\mathbf{Normal}}\left( {{\varvec{\mu}}_{{2,{\varvec{t}}}} ,{\varvec{\varepsilon}}_{2} } \right)$$

$${\varvec{\mu}}_{{2,{\varvec{t}}}} = {\varvec{B}}0 + \user2{B*X}_{{\varvec{t}}}$$

*A0* and *B0* are intercepts, *A* and *B* are vectors of slopes, *X*_*t*_ is the corresponding design matrix holding covariate data, and ε_1_ and ε_2_ correspond to standard deviations. Previous assessments on these last two parameters^55^, as well as preliminary runs of the models here, showed that when estimated they were extremely small and presented large standard errors, therefore they were fixed at 0.001. Standard deviations for modelling location error were derived from Argos error ellipse and calculated as indicated by^56^. Error ellipses were not available for tags deployed before 2013, therefore we generated a model for estimating these based on newer tags data (2016–2019). Gamma regressions were fitted for each location class using calculated standard deviations as observed data and latitude as explanatory variable. Estimated parameters were used for randomly assigning standard deviations values for each location in the pre-2013 data, based on their respective location class and latitude.

**Figure 1:**
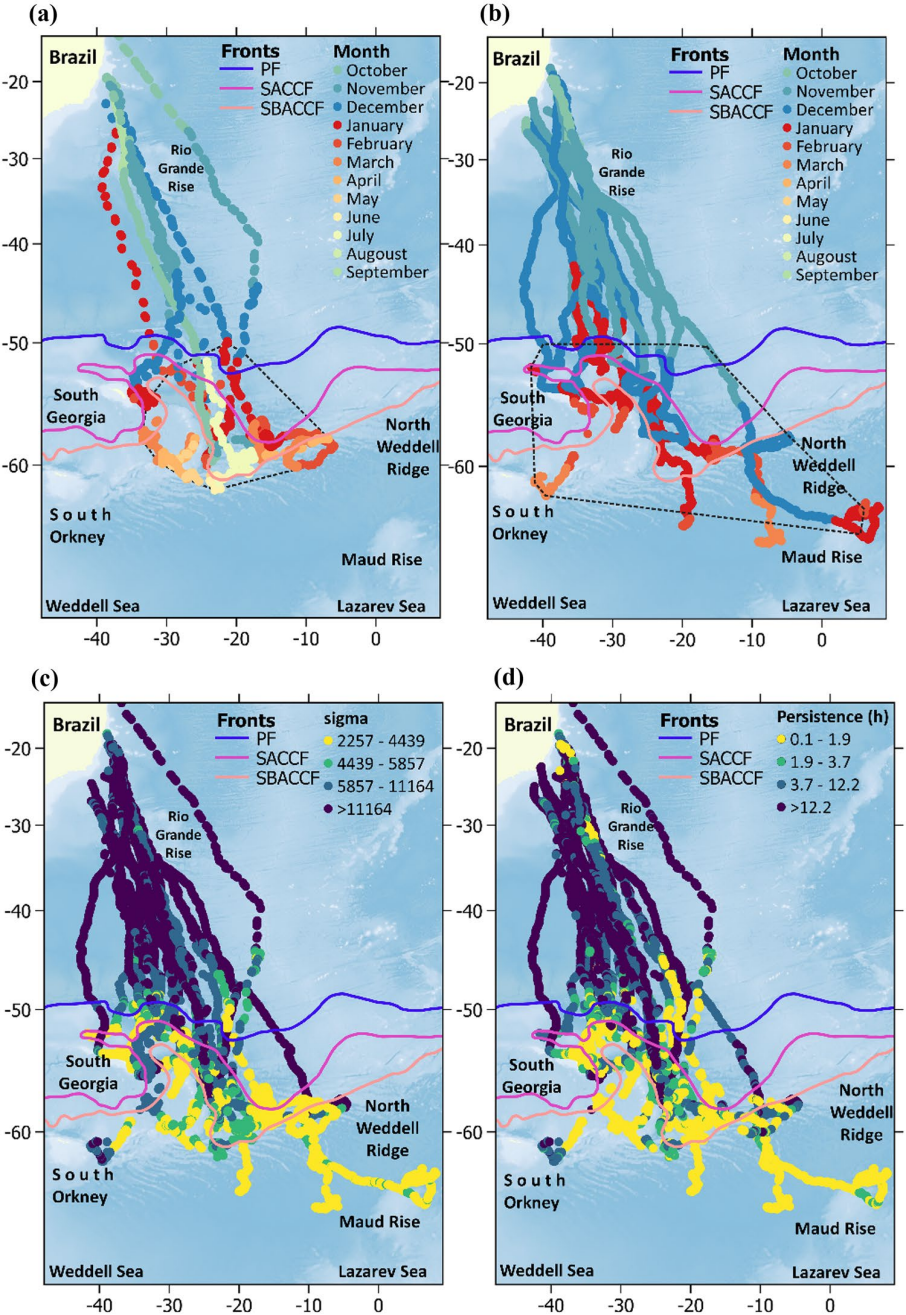
Locations are color coded by month for the first (**a**) and the second (**b**) tagging period. Polygons delimited by black dotted lines indicate the minimum convex polygon for 95% locations south of 50°S. Estimated change in (**c**) sigma *(σ*_*t*_) and (**d**) persistence (*p*_*t*_ = *3/β*_*t*_) for humpback whales based on I-models. Colored lines denote the location of polar front (PF), Southern Antarctic Circumpolar Current Front (SACCF), and the southern boundary of the Antarctic circumpolar current (SBACCF). Antarctic circumpolar fronts data is made publicly available by the Australian Antarctic Data Centre (https://researchdata.edu.au). Data layers (including maps) were created in R ver. 4.0.2 (www.r-project.org) and ensembled in QGIS ver. 3.8.0 (www.qgis.org) for final rendering. Maps were created using data on bedrock topography from the National Centers for Environmental Information (https://maps.ngdc.noaa.gov/viewers/grid-extract/index.html). Grid-cells with values above 0 were considered land coverage and assigned a uniform color.

**Figure 2:**
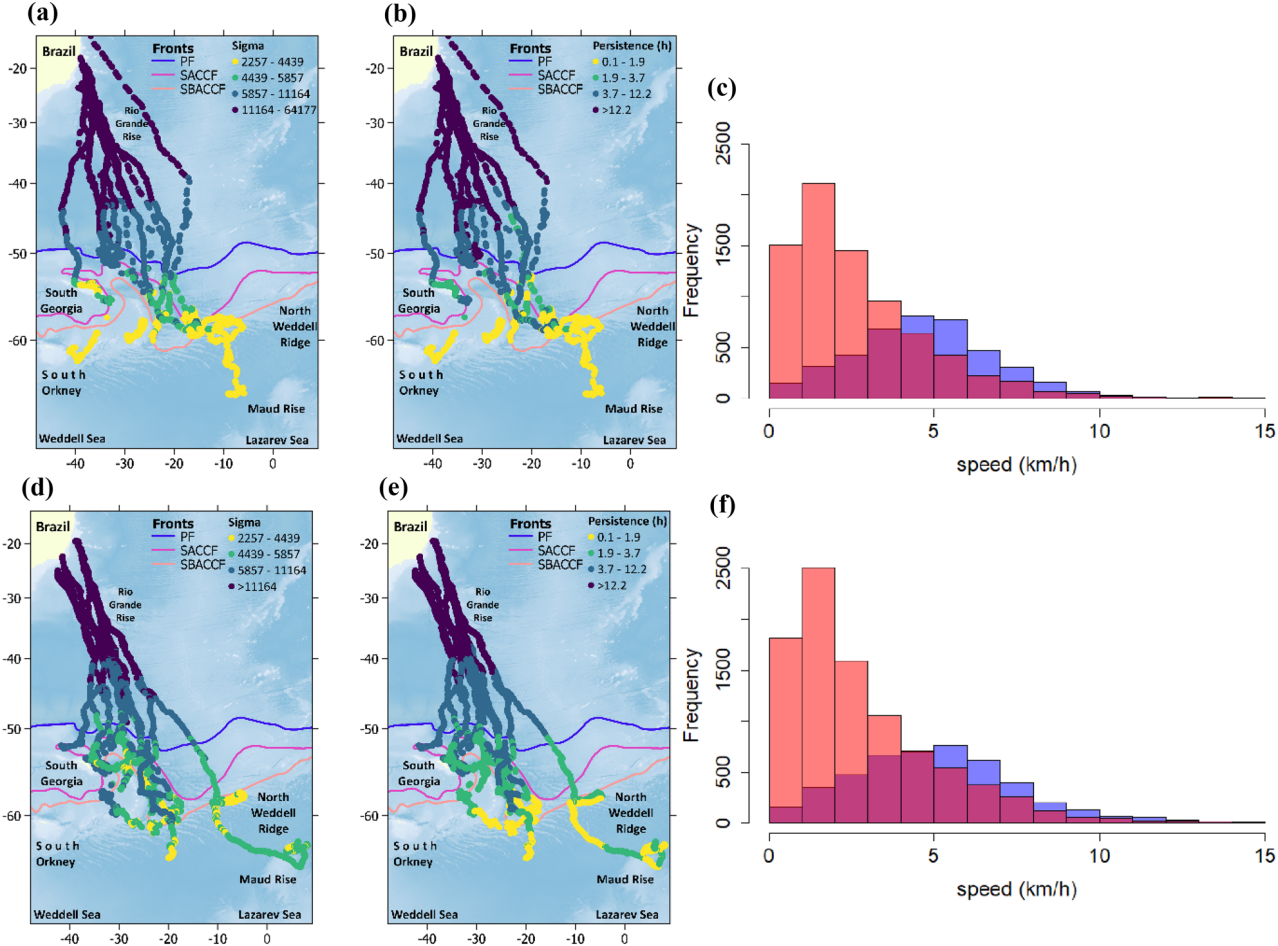
Estimated change in sigma *(σ*_*t*_) and persistence (*p*_*t*_ = *3/β*_*t*_) for humpback whales according to the best P-Model, which included sex differences in parameters controlling movement response to environmental covariates. Overlapping histograms, show speed distribution north (blue) and south (pink) of the 50°S (purple shows the overlap between areas by transparency). Panels a to c show these variables for mothers and panels d to f for males^+^. Colored lines denote the location of polar front (PF), Southern Antarctic Circumpolar Current Front (SACCF), and the southern boundary of the Antarctic circumpolar current (SBACCF). Antarctic circumpolar fronts data is made publicly available by the Australian Antarctic Data Centre (https://researchdata.edu.au). Data layers (including maps) were created in R ver. 4.0.2 (www.r-project.org) and ensembled in QGIS ver. 3.8.0 (www.qgis.org) for final rendering. Maps were created using data on bedrock topography from the National Centers for Environmental Information (https://maps.ngdc.noaa.gov/viewers/grid-extract/index.html). Grid-cells with values above 0 were considered land coverage and assigned a uniform color.

Covariate data were standardized (centered and scaled), and missing values (only occurred for CHL) were filled with the previous location value on each whale. Our modelling approach allowed us to depict the influence of environmental covariates on *β*_*t*_ and *σ*_*t*_ , with higher values of *σ*_*t*_ indicating higher velocities and higher values of *β*_*t*_ indicating lower directional persistence, which might be expressed as *p*_*t*_ = 3/*β*_*t*_ in units of time^54^. In other words, *p*_*t*_ indicates the amount of time separating two sets of locations and speed for them to be uncorrelated. In this manner, ARS is characterized by lower values of *p*_*t*_ and *σ*_*t*_ in opposition to transit (higher values of *p*_*t*_ and *σ*_*t*_). Although ARS state cannot be discretely estimated through this approach, we separated *p*_*t*_ and *σ*_*t*_ values along whales’ tracks in 25% percentiles, so the lowest quartiles could be regarded as ARS behavior (Figs. 1, 2).

This model was fitted to data from each individual whale separately (I-model), but we also analyzed the entire data set jointly, assuming complete pooling of parameters (P-model). P-model assumes all whales respond similarly to environmental variables and allows borrowing statistical strength for parameters estimation^30^. However, for assessing the effect of grouping whales by sex or tagging period, we modified the equations above to incorporate additional parameters controlling deviations for intercepts and slopes for one group against the other.

$${\varvec{\mu}}_{{1,{\varvec{t}}}} = \left( {{\varvec{A}}0 + {\varvec{a}}0{\varvec{G}}_{{\varvec{t}}} } \right) + \left( {{\varvec{A}}_{1} + {\varvec{a}}_{1} {\varvec{G}}_{{\varvec{t}}} } \right)\user2{*X}_{{1,{\varvec{t}}}} + \cdots + \left( {{\varvec{A}}_{{\varvec{n}}} + {\varvec{a}}_{{\varvec{n}}} {\varvec{G}}_{{\varvec{t}}} } \right)$$

$${\varvec{\mu}}_{{2,{\varvec{t}}}} = \left( {{\varvec{B}}0 + {\varvec{b}}0{\varvec{G}}_{{\varvec{t}}} } \right) + \left( {{\varvec{B}}_{1} + {\varvec{b}}_{1} {\varvec{G}}_{{\varvec{t}}} } \right)\user2{*X}_{{1,{\varvec{t}}}} + \cdots + \left( {{\varvec{B}}_{{\varvec{n}}} + {\varvec{b}}_{{\varvec{n}}} {\varvec{G}}_{{\varvec{t}}} } \right)$$

Alternative P-models were constructed using all possible combinations of covariates without simultaneously incorporating two or more strongly correlated covariates. Pearson Correlation analyses were used to depict such correlations (*r* ≥ 0.5, *p* < 0.01) yielding high correlations for SST, CHL and DSB, therefore competing models never considered these covariates simultaneously among possible combinations. Akaike Information Criterion (AIC)^57^ was used to select the most parsimonious (lowest AIC value) model. If large individual variability exists among whales, complete pooling of parameters would yield averages that might not be representative of neither of the existing variations. Therefore, once the best P-model was selected, I-models’ predictions on *β*_*t*_ and *σ*_*t*_ responses to selected covariates were visually compared to those of the best P-model. ICE08 and ICE10 could not be used to fit I-models for some whales that never visited areas of sea ice coverage during August or October (Tables 1 and S1).

Finally, best P-model results were used to generate spatial predictions for *p* and *σ* using a 0.1 × 0.1 degrees grid. These predictions indicate the expected behavioral responses for whales traversing areas not necessarily visited during the tracking period. Predictive layers were generated using selected oceanographic conditions from January (when most migrating whales are expected to have arrived to feeding grounds) during the previous 10 years (2010–2019, Figs. S2–S4) since the last tagging campaign and averaged as a manner to considerate environmental interannual variation.

All models were fit using Template Model Builder (TMB), a R package that relies on the Laplace approximation combined with automatic differentiation to fast-fit models with latent variables^58–60^.

### Statement of approval

This research was authorized by research permits provided by the Government of Brazil (Conselho Nacional de Desenvolvimento Cientifico e Tecnológico [CNPq, #CMC 026/02-028/03], and Ministério do Meio Ambiente [IBAMA, permit #009/02/CMA, IBAMA, process #02,001.000085/02-27, ICMBio #11,523-1 and, SISBIO #53,354-4, ABIO 857/2017, ABIO 1149/2019]). All methods were performed in accordance with the national guidelines and regulations. Additionally, the methods employed in this study were consistent with those approved by the Institutional Animal Care and Use Committee of the National Marine Mammal Laboratory of the Alaska Fisheries Science Center, National Marine Fisheries Service, U.S. National Oceanic and Atmospheric Administration. This study is reported in accordance with ARRIVE guidelines.

## Results

### Migratory pathways

The onset of migratory behavior ranged between late September to late December, with most of the whales departing Brazilian coastal waters in October (Table 2). With one exception, all whales departed the continental shelf off central Brazil between 19° and 24° S, and 36° and 42° W, just south of the species main breeding habitat, the Abrolhos Bank (Fig. 1). These whales initially migrated showing high values of *p*_*t*_ and *σ*_*t*_, following a relatively narrow migratory corridor (about 600 km wide) up to approximately 30–31° S, where they reached the Rio Grande Rise (RGR). At the RGR, a noticeable reduction in *p*_*t*_ and *σ*_*t*_ was observed for some whales, before the migratory pathways are expanded latitudinally as whales continue their migration south towards high latitudes in the SWA (Fig. 1). One individual, ID2009-87,783, departed from the NE coast of Brazil (at approximately 15° S and 36° W) and migrated towards the Mid-Atlantic Ridge (at about 40° S, Fig. 1), where it remained for nearly all December, before migrating S-SW towards the South Sandwich Islands (SSI). Low values of *p*_*t*_ and *σ*_*t*_ were documented in this region, suggesting this individual may have used this area for purposes other than migration (Fig. 1).

**Figure 3:**
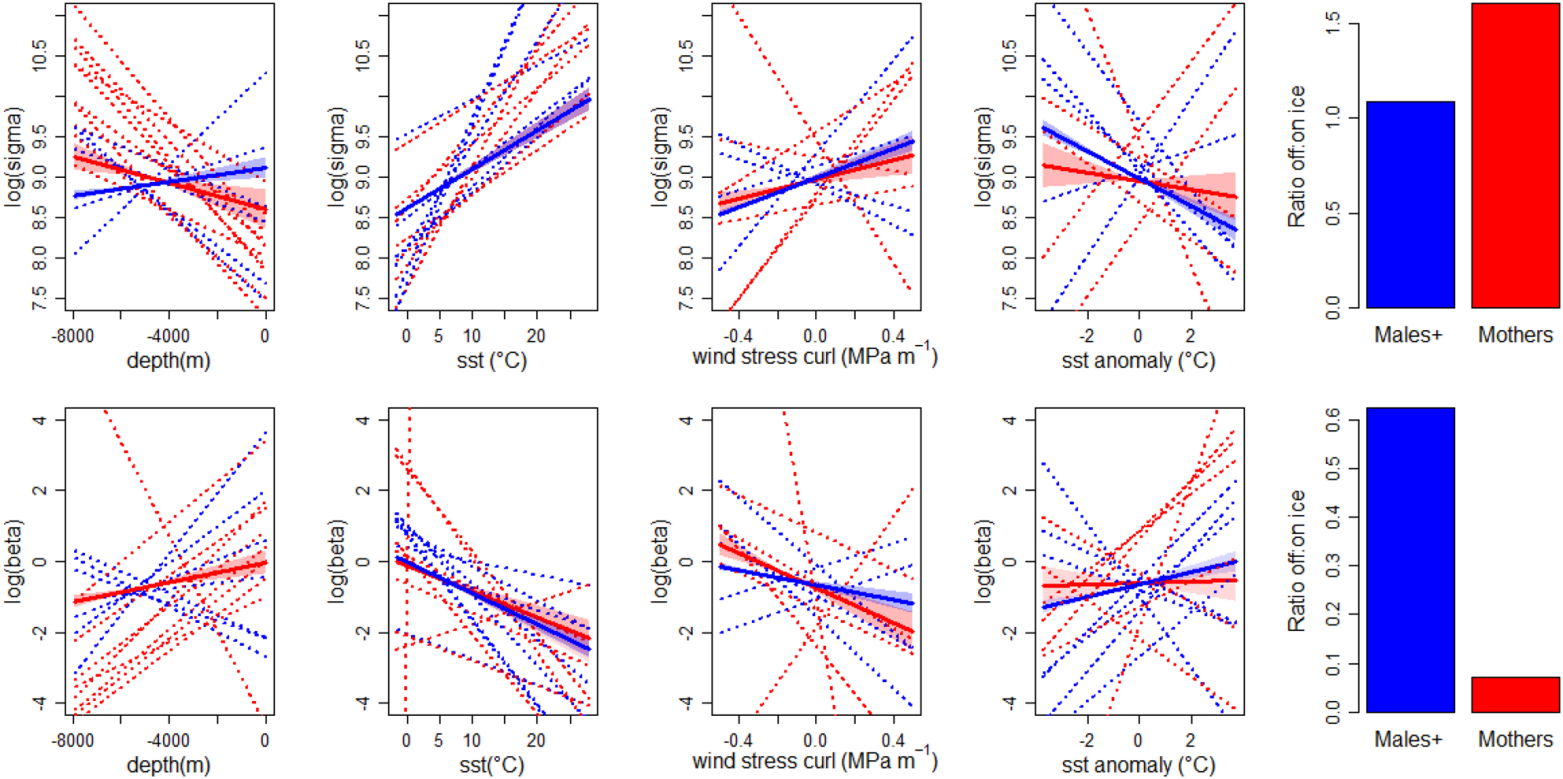
Thick lines indicate the predicted effect of environmental covariates on sigma (*σ*, top row) and beta (*β*, bottom row) in the log scale for mothers (red) and males^+^ (blue), based on the best population model (P-model). Shaded areas indicate 95% confidence intervals. Thin dotted lines indicate individual model (I-models) predictions for each tagged whale. Bar plots indicate the effect of ice on *σ* and *β* as the ratio between the intercepts for the linear predictors and the intercepts plus the effect of the binary variable ICE08.

Migratory displacement duration (time required to move from Brazilian shelf to the approximate location of Polar Front at 50° S) ranged from 25 to 71 days (mean = 37.6, median = 35, SD = 13.9, Table 2). When sex and tagging period were considered, mothers migration ranged from 28 to 71 days (mean = 41, median = 38, SD = 13), males^+^ migration ranged from 25 to 50 days (mean = 34, median = 31, SD = 9), migration in the first tagging period ranged from 28 to 71 days (mean = 46, median = 38, SD = 15), and migration in the second tagging period ranged from 25 to 50 days (mean = 33, median = 34, SD = 7). Estimated speed ranged between 0.001 to 32 km/h (mean = 3.5 km/h, median = 2.9 km/h, SD = 2.5), although 99.99% of these ranged between 0.001 and 15 km/h. When sex, and latitude were considered, mothers speed north of °50S (0.05–32, mean = 4.7 km/h, median = 4.7, SD = 2.2) was slower than males^+^ (0.1–17, mean = 5.1 km/h, median = 4.9, SD = 2.5) and this difference was statistically significant (Wilcoxon rank sum test W = 10,430,084, *p* =  < 0.001). South of 50°S both groups showed similar speeds (females, 0.001–22, mean = 2.7 km/h, median = 2.1, SD = 2.2 and males^+^, 0.01–18.7, mean = 2.8 km/h, median = 2.1, SD = 2.2, Wilcoxon rank sum test W = 35,435,991, *p* = 0.1192, Fig. 3).

### Habitat use in foraging grounds

Whales migrated towards feeding destinations at and to the west of the Scotia Sea (SS), including nearshore waters off South Georgia (SG), the South Orkney Islands (SO), and the South Sandwich Islands (SSI), and the northern Weddell and Lazarev Seas. Telemetry data suggests that the feeding habitats include a 50° longitudinal section of the South Atlantic Ocean, from about 10° E to 40° W between the Polar Front and latitudes as far south as 65° S (Figs. 1 and 2).

The first signs of ARS in the feeding grounds were observed around or south of the Polar Front (Figs. 1 and 2). ARS locations were seen close to shore, for example to the north of SG, west of the SSI and west of the SO, while offshore ARS locations were typically associated with the North Weddell Ridge along the Southern Antarctic Circumpolar Current Front (SACCF) and into the Weddell and Lazarev Seas (Fig. 1). For the latter area on male whale (ID2018-172,008) reached Maud Rise, which constitute the most southeastern location reached by any of the tagged whales so far (Fig. 1b). Ten whales never visited areas covered by sea ice during August and/or October and five others did it only briefly (less than 20% of the tracking data for one or both months considered in the analysis, Table 2). Pearson Correlation analyses showed a positive significant correlation (r ≥ 0.5, *p* < 0.01) between individual tracking duration and the percentage of the data associated with sea ice cover during August and October.

A distinct pattern in habitat use in the feeding grounds was observed between the two tagging periods. Animals tagged between 2003 and 2012 remained in the SS, east of SG, as far west as 5° W and typically north of 60° S (Fig. 1a). In contrast, whales tagged between 2016 and 2019 showed a broader use of the feeding grounds in the southern South Atlantic, including areas west of SG (at about 40° W), east towards the 10^o^E meridian, and into the northern Weddell Sea and Lazarev Sea (Fig. 1b). The minimum convex polygon (MCP) for 95% of the satellite locations within the feeding grounds were 1,419,039 km^2^ and 3,556,347 km^2^ for whales instrumented in 2003–2012 and 2016–2019, respectively (Fig. 1a,b).

Results for the top five most supported P-models according to AIC are presented in Table 3. The best P-model retained DEPTH, SST, CURL, SSTA and ICE08 as explanatory variables (Table 3). The inclusion of additional parameters controlling group mediated differences (sex and tagging period) on covariate parameters improved models´ fit, with those associated to sex yielding the best results. In general, the signs of the responses to environmental covariates were the same between mothers and males^+^, except for DEPTH, with mothers reducing *p*_*t*_ (increasing *β*_*t*_) and *σ*_*t*_ at shallower waters and males eliciting an opposite trend for *σ*_*t*_ with no significant effect on *β*_*t*_ (Fig. 3). This model indicates that whales tended to reduce their velocity and movement persistence (ARS behavior) at colder waters where wind-induced upwelling is likely to occur (negative CURL values in Southern Hemisphere), SST anomalies are positive and sea ice was present in August in the year each individual was tagged (Fig. 3, Table 3).

**Table 3:** Results for the five best *P*-models.

Model	B0		b0		B1		b1		B2		b2		B3		b3		B4		b4		B5		b5		ΔAIC
	PC	SE	PC	SE	PC	SE	PC	SE	PC	SE	PC	SE	PC	SE	PC	SE	PC	SE	PC	SE	PC	SE	PC	SE	
DEPTH + SST + CURL + SSTA + ICE08 + SEX	**− 0.60**	**0.03**	**− 0.12**	**0.05**	**− **0.01	0.03	**0.18**	**0.04**	**− 0.68**	**0.03**	**0.11**	**0.05**	**− 0.17**	**0.03**	**− 0.23**	**0.04**	**0.13**	**0.02**	**− 0.11**	**0.03**	**0.47**	**0.08**	**2.17**	**0.15**	0.0
DEPTH + SST + CURL + SSTA + ICE10 + SEX	**− 0.61**	**0.03**	0.04	0.05	**0.13**	**0.04**	0.14	0.05	**− 0.67**	**0.03**	0.05	0.05	**− 0.15**	**0.03**	**− 0.25**	**0.04**	**0.14**	**0.02**	**− **0.07	0.04	**0.54**	**0.09**	**1.94**	**0.16**	180.4
DEPTH + SST + CURL + SSTA + ICE10 + PERIOD	**− 0.5**	**0.06**	**− **0.11	0.07	**0.21**	**0.06**	**− **0.11	0.07	**− 0.45**	**0.05**	**− 0.32**	**0.05**	**− 0.43**	**0.04**	**0.32**	**0.05**	**− **0.06	0.04	**0.25**	**0.04**	**1.93**	**0.13**	**− 0.80**	**0.17**	224.8
DEPTH + SST + CURL + SSTA + ICE08 + PERIOD	**− 0.58**	**0.05**	**− 0.12**	**0.06**	**0.46**	**0.06**	**− 0.37**	**0.07**	**− 0.40**	**0.05**	**− 0.30**	**0.06**	**− 0.45**	**0.04**	**0.34**	**0.05**	**− 0.09**	**0.04**	**0.28**	**0.04**	**1.94**	**0.12**	**− 0.31**	**0.16**	229.7
DEPTH + SST + CURL + SSTA + ICE08	**− 0.66**	**0.03**			**0.12**	**0.02**			**− 0.57**	**0.02**			**− 0.30**	**0.02**			**0.07**	**0.02**			**1.71**	**0.07**			686.0

Model	A0		a0		A1		a1		A2		a2		A3		a3		A4		a4		A5		a5		**ΔAIC**
	PC	SE	PC	SE	PC	SE	PC	SE	PC	SE	PC	SE	PC	SE	PC	SE	PC	SE	PC	SE	PC	SE	PC	SE	
DEPTH + SST + CURL + SSTA + ICE08 + SEX	**8.94**	**0.02**	**− **0.02	0.02	**0.06**	**0.02**	**− 0.17**	**0.02**	**0.37**	**0.02**	0.06	0.03	**0.15**	**0.01**	**− 0.05**	**0.02**	**− 0.13**	**0.01**	**0.09**	**0.02**	**− 0.08**	**0.03**	**− 0.39**	**0.04**	0.0
DEPTH + SST + CURL + SSTA + ICE10 + SEX	**8.95**	**0.02**	**− **0.04	0.02	**0.06**	**0.02**	**− 0.16**	**0.02**	**0.36**	**0.02**	0.06	0.03	**0.14**	**0.01**	**− 0.05**	**0.02**	**− 0.13**	**0.01**	**0.09**	**0.02**	**− 0.12**	**0.03**	**− 0.40**	**0.04**	180.4
DEPTH + SST + CURL + SSTA + ICE10 + PERIOD	**9.17**	**0.03**	**− 0.30**	**0.03**	**− **0.01	0.02	**− 0.06**	**0.02**	**0.16**	**0.02**	**0.35**	**0.03**	**0.14**	**0.01**	**− 0.11**	**0.02**	0.04	0.01	**− 0.19**	**0.02**	**− 0.81**	**0.04**	**0.65**	**0.05**	224.8
DEPTH + SST + CURL + SSTA + ICE08 + PERIOD	**9.10**	**0.02**	**− 0.21**	**0.03**	**− 0.08**	**0.01**	0.02	0.02	**0.20**	**0.02**	**0.27**	**0.03**	**0.17**	**0.01**	**− 0.13**	**0.02**	**0.03**	**0.01**	**− 0.19**	**0.02**	**− 0.63**	**0.04**	**0.34**	**0.05**	229.7
DEPTH + SST + CURL + SSTA + ICE08	**8.92**	**0.01**			**− 0.06**	**0.01**			**0.37**	**0.01**			**0.13**	**0.01**			**− 0.09**	**0.01**			**− 0.34**	**0.02**			686.0

Maximum likelihood estimate for parameter coefficients (PC) and the corresponding standard error (SE) are provided. Significant parameter estimates (*P* ≤ 0.05) are denoted in bold. Values for **ε**_**1**_ and **ε**_**2**_ were fixed at 0.001.

Parameter estimates for each I-model are provided as supplementary information in Table S1. In general, I-models were consistent with P-models’ overall trend regarding the effect of SST and ICE08 on *σ*_*t*_ and *β*_*t*_ (Fig. 3, Table S1). Mothers’ individual predictions were more coherent with P-model´s output, ranging between zero and three individuals showing an opposite sign in covariate parameters related to *β*_*t*_ (DEPTH = 1, SST = 2, CURL = 2, SSTA = 3 and ICE08 = 0) and ranging between zero and two for *σ*_*t*_ (DEPTH = 0, SST = 0, CURL = 2, SSTA = 2 and ICE08 = 2). Males + presented more variation ranging between zero and three individuals showing an opposite sign in covariate parameters related to *β*_*t*_ (SST = 0, CURL = 2, SSTA = 3 and ICE08 = 0) and between zero and four for *σ*_*t*_ (DEPTH = 4, SST = 0, CURL = 2, SSTA = 2 and ICE08 = 1). The effect of DEPTH on *β*_*t*_ for Males + was not significant in the P-model but showed a negative effect in three individuals and a positive effect in 4 individuals when considering I-models (Fig. 3, Table S1).

**Figure 4:**
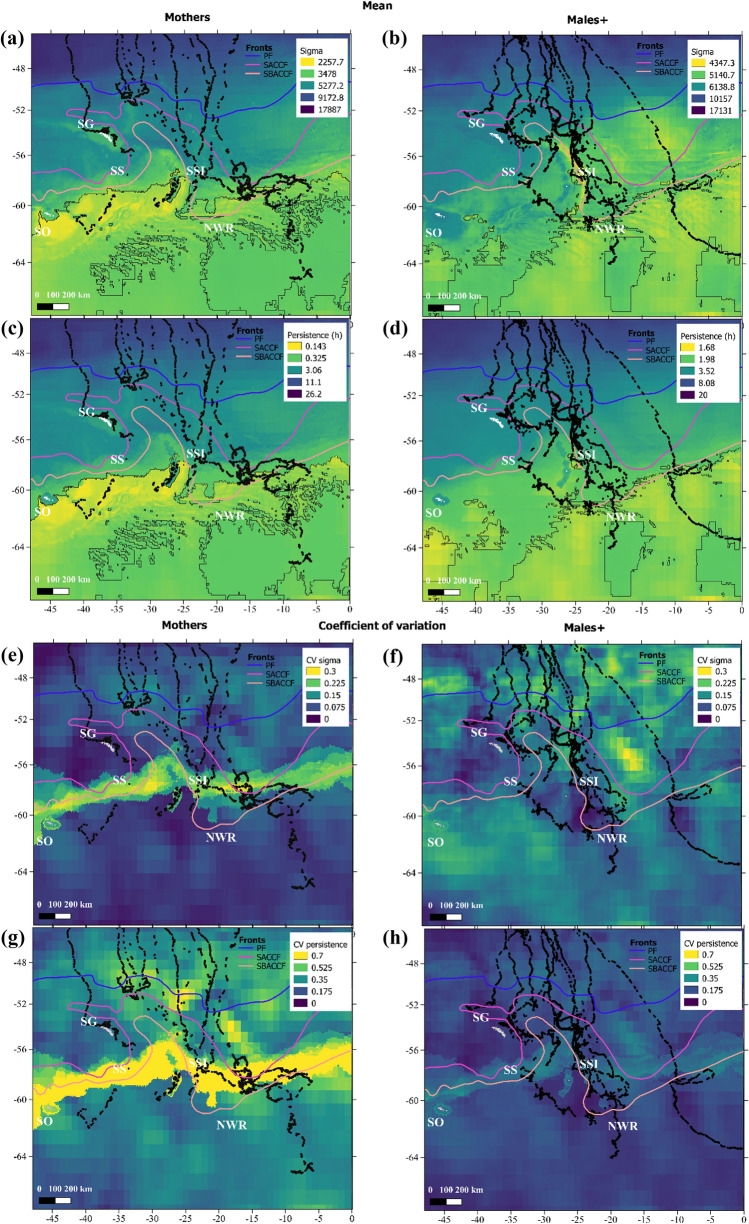
Top panels (**a**–**d**) show mean expected change in sigma (*σ*, **a**,**b**) and persistence (*p*_*t*_ = *3/β*, **c**,**d**) based on oceanographic conditions of January from 2010 to 2019, using the best P-model. Colors in spatial predictions are based on 25% percentiles. Black contoured polygons indicate areas where the 25% lowest values of sigma and persistence cooccurred. Bottom panels (**e**–**h**) show associated coefficient of variation for the means. Left panels (**a**, **c**, **e**, **g**) show results for mothers and right panels show results for males^+^ (**b**, **c**, **g**, **h**). Black dots represent observed locations at the feeding grounds. Colored lines denote the location of polar front (PF), Southern Antarctic Circumpolar Current Front (SACCF), and the southern boundary of the Antarctic circumpolar current (SBACCF). The location of South Georgia (SG), Scotia Sea (SS), South Sandwich Islands (SSI), South Orkney (SO) and Northern Weddell Ridge (NWR) are indicated by respective acronyms. Antarctic circumpolar fronts data is made publicly available by the Australian Antarctic Data Centre (https://researchdata.edu.au). Data layers were created in R ver. 4.0.2 (www.r-project.org) and ensembled in QGIS ver. 3.8.0 (www.qgis.org) for final rendering.

Results from the best P-model also showed a considerable larger effect of ICE08 on *β*_*t*_ and *σ*_*t*_ for mothers in comparison to males + . Considering the ratio $\frac{{e}^{({A}_{0}+{a}_{0}G)}}{{e}^{{((A}_{0}+a0G)+{(A}_{5}+{a}_{5}G)*ICE08)}}$ males + and mothers using areas free of ice during the preceding winter showed that the average expected *σ* was 1.08 and 1.61 times those observed in areas covered by ice respectively. Considering the ratio $\frac{{e}^{({B}_{0}+{b}_{0}G)}}{{e}^{{((B}_{0}+b0G)+{(B}_{5}+{b}_{5}G)*ICE08)}}$ males + and mothers using areas free of ice during the preceding winter showed that the average expected *β* was 0.625 and 0.07 times those observed in areas covered by ice respectively. This resulted in spatial predictions for mothers’ *p* and *σ* on feeding grounds were considerably lower than those expected for males + (Fig. 4a–d). In general, both groups were expected to show the lowest values of *p* and *σ* around and south of 60°S. However, mothers were expected to concentrate more to the west and males + more to the east with some overlapping areas (Fig. 4a–d). Coefficient of variation (CV) from mean predictions of *p* and *σ* (2010–2019) showed that the highest variability on models’ spatial predictions were concentrated around 60°S matching the interannual variation in the winter ice northern boundary (Fig. 4e–h). CV for *σ* in the case of males + differed from this pattern showing larger variation north of 56°S (Fig. 4f).

## Discussion

The migratory routes and destinations documented in this study confirms the areas around the SS represent an important feeding ground for SWA humpback whales. Previously, migratory connectivity was established by satellite tracking (including some of the tracks used in the present analysis^7,41,42^ and photo-identification data^38,40,61^ showing whales used habitats close to SG and the SSI. This study adds new migratory connections for humpback whales wintering off Brazil, including foraging areas near the SO, the South-Scotia and North Weddell ridges, Weddell Sea, and the Maud Rise/Lazarev Sea (Figs. 1 and 2).

Movement models showed that most of the whales reduced *p*_*t*_ and *σ*_*t*_ (ARS) south of the Polar Front (~ 50°S) with few signs of using stopover areas during migratory displacements. Exceptions from this pattern were observed by some whales eliciting ARS in some areas north of 50°S (Fig. 1). Clear examples include a male (ID2012-121,189) at the RGR and a mother (ID2009-87,783) at the Mid-Atlantic Ridge (Fig. 1c,d). It is difficult to determine whether these whales were opportunistically exploiting suitable foraging conditions outside their main foraging grounds, using alternative migratory pathways, or both. However, the use of rises and seamounts has been reported for other humpback whale population in the Southern Hemisphere and hypothesized to represent possible alternative breeding grounds, feeding habitats, and/or navigational landmarks^21,62^. Besides the aforementioned discrepancies, the latitude of ~ 50°S appears to represent an appropriate northern boundary for SWA humpback whales feeding grounds. Inspecting migratory displacement times and speed south and north of this boundary showed that mothers took more time to reach it and travelled slower than males^+^. This was expected as females accompanied by calves have been reported to migrate at slower speeds, alternating between active swimming and resting/nursing activities^63^. However, these results should be considered with caution as calf mortality is relatively high in humpback whales (*e.g.*, approximately 20% of calves die within the first 6 months of the year^64^), suggesting that some of the mothers tagged in the presence of a calf may not have been accompanied by their offspring at the time they migrated or during the whole migration. South of 50°S speed was overall lower for both sex classes with no signs of differences between them (Fig. 2).

Our modelling framework allowed us to inspect how complete pooling on parameters estimations across individuals differed from fits using individual data only. In general, results from the best P-model (which included sex grouping, Table 3) showed that ARS was associated to colder waters following the latitudinal gradient from the Brazilian coast to the southern South Atlantic Ocean (Table 3, Fig. 3, S2). Results from I-models were highly consistent with P-models results in this sense (Fig. 3, Table S1), which indicates that SST might be regarded as a reliable and coarse indicator on the probability of initializing foraging behavior south of the Polar Front (~ 50°S). Interestingly, movement parameters of whale #ID 2018-172,008 (Table S1) were not significantly correlated with SST. Contrary to most whales, this male followed mostly a directional movement pattern since it departed from Brazilian coast, presented signs of ARS over the North Weddell Ridge, and then headed southeast until it reached Maud Rise, Lazarev Sea, before transmission ceased. ARS for this individual whale was present south of ~ 60°S (Figs. 1 and 2), henceforth, the large amount of time engaged in transit behavior south of 50°S yielded SST irrelevant for describing movement patterns for this whale.

ARS was also associated with shallower waters (Fig. 3, Table 3) at shelf and shelf-break areas around SG, the SO and the SSI, which are recognized as a high nutrient, high chlorophyll ecosystem^18,65,66^ where high krill biomass occur^67–70^. Although other offshore areas over the North Weddell Ridge where ARS was also predominant are substantially deeper, they are still relatively shallower than the very deep waters (> 2000 m) where transit-like behavior was performed (Figs. 1 and 2). Considering sex, models showed that mothers were mainly responsible for this type of response, with males^+^ eliciting an opposite mild response, preferring deeper offshore areas (Fig. 3). Results from I-models showed that only four males were responsible for this effect, with the rest of the whales preferring shallower waters in a broad sense (Fig. 3, Table S1). The preference for a large bathymetric range, specially but not restricted to males^+^, could be explained by whales selecting on-shelf foraging areas at SG, SO, SSI, as well as waters downstream of the ACC over the North Weddell Ridge (Fig. 1). Although some whales migrated directly into the Northern Weddell Ridge, others visited the SS first and then moved to the former, reflecting the capability of humpback whales to alternate between these two types of habitats during feeding season. ACC plays a major role in nutrient supply, with phytoplankton blooms that can be traced up to 3,500 km east of SG over the Northern Weddell Ridge^71^. ACC is also crucial for krill dispersal and retention^72–74^ generating suitable foraging conditions for humpback whales at offshore areas east of the SS. Even when it was not retained in the best models, ARS was also associated with lower values of DSB, matching the flow of ACC in general, but also, the northern boundary of ice cover during preceding months of whales’ arrival.

Whales where more likely to elicit ARS behavior at zones that were occupied by ice during winter and spring of each year, with P-models yielding better fitting when using winter ice coverage (ICE08, Table 3). Sea ice extent is considered important for explaining krill distribution^75^ as it provides food supply (with large spatiotemporal and ontogenetic variability) and shelter during winter^69,76–78^. It is also an important source of iron supply for phytoplankton blooms during melting season^79^. Although, the importance of sea ice cover and retreat timing on krill distribution, recruitment and spawning is not unequivocal^80–82^, the marginal ice zone is thought to be particularly relevant for these processes^77,80,83^. Henceforth, it represents an important area for securing prey resources for humpback whales^20,22,84–87^. How ice modulates Humpback whales’ distribution, however, differs among distinct regions^86^. For some areas whales occur in proximity to ice edge during summer^84,87^ while in others they occur in areas where ice retreated in a time-scale of months^20,22^. Among whales tagged in this study, those tracked for more time tended to present more locations with presence of ice during winter and spring (Table 1) suggesting whales start foraging in areas that were never covered with ice (*i.e.,* SG, areas around PF) with a subsequent southward progression in foraging activities.

A preference for positive daily SSTA anomalies could be explained by assuming the overall rapid warming pattern in SS and surrounding waters^88^. An inspection of the spatial patterns of SSTA within the study area from 2010 to 2019 revealed that at times certain areas were more homogeneously warmer, however, for other areas SSTA shows spatial patterns of rounded shapes (Fig. S4) consistent with the occurrence of oceanographic eddies^89^. Cyclonic eddies tend to present colder cores as they upwell deep cold water rich in nutrients, thus enhancing productivity^90^. Recent findings suggest, however, that anticyclonic eddies (with warmer core) during late summer and autumn might be even more productive than cyclonic eddies because they permit deeper water mixture during preceding months, hence allowing more nutrients to enter their interiors^89^. In either case, eddies are known to concentrate krill at their cores or margins^91–93^ providing suitable feeding grounds for upper trophic species^94^. Results from I-models showed that the response to SSTA elicited the largest inter-individual variation, with whales having almost the same number of individuals with positive and negative correlations with movement parameters (Fig. 3, Table S1). This suggests that whales might be responding to mesoscale features, however, the case of an overall warming cannot be ruled out (see later) and will require further scrutiny. A preference for negative values of CURL is consistent with their preponderance south of the polar front^95,96^. Negative CURL values promote Ekman pumping and wind induced upwelling, however, its importance on primary productivity is more likely to be relevant over long time scales rather than on a daily basis^95,97^. Time lags between CURL patterns and krill distribution have been observed^98^ hence this type of lagged variable could be further explored for understanding whales’ movement pattern.

Individual variation in movement patterns can arise from real differences on habitat characteristics selected by humpback whales, or from sampling limitations derived from tracking duration. For instance, four males + and five mothers showed no significant correlation with at least one movement parameter and SST, and for two mothers it presented and opposite sign to what P-models showed (Fig. 3, Table S1). In four of these cases, whales provided with little amount of data south of the PF before transmission ceased, therefore when analyzing them individually, covariate parameters were mostly reflecting variation elicited during migration. The opposite was true for another case where the whale only presented data over the Northern Weddell Ridge, hence no data associated with warmer waters were available. An extreme case occurred on those whales which never reached areas covered with sea ice making unfeasible to evaluate the effect of ICE08/10 on their movement parameters. These examples indicate that the complete pooling approach of P-models provides an efficient tool for improving movement parameters estimates, partially overcoming the large variation in tracking duration, by integrating information from multiple individuals. However, males + movement patterns appear to be subjected to more interindividual variability; this might just arise from unaccounted factors such as age class or missing information (*i.e.,* undetermined sex) and should be addressed in the future as more data becomes available.

Considering large-scale ecosystem modification undergoing at Southern Ocean, we aimed to assess how whales’ movements patterns responded to environmental conditions there, as a steppingstone towards understanding how changes might affect this population. An interesting aspect arising from the migratory patterns described here was the incursion of whales into the Weddell and Lazarev Seas during the second tagging period (2016–2019). Animals tracked between 2003 and 2012 (n = 8, Table 1) remained relatively further to the north, barely crossing 60^o^S (Fig. 1). During the second tagging period, at least three whales (20% of the animals tagged in the second period) moved further to the south, reaching latitudes of 65^o^S (Fig. 1b). When tagging period was considered as a factor contributing to differences in covariate parameters, model fitting was improved compared to models not considering whale grouping, and only outweighed by those models grouping by sex (Table 3). It should be considered though, that during the first tagging period six out of eight tracked whales were mothers, making impossible to separate the effect of sex from period grouping. P-models considering tagging period showed that whales responded similarly to DEPTH between periods decreasing *p*_*t*_ and *σ*_*t*_ values at shallower waters, and that the effect of SST was greater during the second period (Table 3). Response to ICE08/10 indicated a larger probability of reducing *p*_*t*_ and *σ*_*t*_ values in areas previously covered with ice during the first period (Table 3), agreeing with mothers being largely responsible for this pattern and the fact that during the second period very few whales visited areas previously covered with ice (Table 1). Finally, a significant (negative) correlation was found between *p*_*t*_ and *σ*_*t*_ and SSTA only for the second period, which was accompanied by a weaker positive correlation with CURL (Table 3). Our results are coherent with the 2015–2018 historical record decrease in sea ice coverage observed in the Southern Ocean, including the Weddell and Lazarev Seas, when positive SSTA and CURL anomalies were observed^99,100^. Even when the sample size presented in this study is limited and the effect of sex cannot be separated from period grouping, the present results suggest that some humpback whales might have responded to modifications in their habitat during the second tagging period by using more southernly waters. Exploiting the western part of the Lazarev Sea might not be the most rewarding as krill biomass is significantly lower than in the SS^69,101^. Unlike the latter, Lazarev sea is characterized by shorter bloom periods due to a larger portion of the year covered with ice^69,101^. However, whale #ID 2018–172,008 reached the eastern part of Maud Rise where a recurrent polynya has been recorded since the 70 s, reaching its maximum size on 2017^102,103^. Local high krill biomasses and large predator aggregations have been reported to occur in this area^101,104,105^ suggesting this could represent an important, potentially novel, feeding area. Even when limitations on tracking duration could hinder our view on the extent to which waters south 60ºS are used by humpback whales, it should be considered that the three individuals tracked the longest (#ID 2012-121,189, 2012-111,871 and 2003-24,642, Table 2) remained between the SS and the Northern Weddell Ridge and that all these whales were tagged in the first period. In addition to the described scenario there is evidence that the Antarctic krill (*Euphausia superba*) has been contracting southward in the South Atlantic^17^ in response to warming environmental conditions. Henceforth, results presented here suggest that humpback whales may be showing early signs of a shift in distribution or an expansion of their feeding grounds in high latitudes of the South Atlantic, as has been reported for other top predator species in response to climate change elsewhere^106,107^.

Using P-model results to generate spatially explicit predictions of how humpback whales might respond to oceanographic conditions showed that areas around and south of 60°S are important for this population (Figs. 4, S1). Predictions for both groups highlighted the South-Scotia and North Weddell ridges, as well as SSI as the main areas for ARS which is consistent with the data. However, many areas to the south were also highlighted as likely to host ARS with large differences between groups (Fig. 4). These differences arising from estimated parameters should be considered with caution specially for males + . As described above the influence of a few individuals altered the general pattern of how whales responded to DEPTH and large interindividual variability was observed for SSTA and CURL. In addition, large interannual differences in oceanographic conditions pose a crucial role in our spatial predictions. Using the coefficient of variation on the spatial predictions based on data from 2010 to 2019 showed that the area surrounding the ice-edge concentrated the largest variation, followed by some areas north of this (Fig. 4e–h). This indicates that the environmental conditions selected by humpback whales are more common and more predictable around and south of 60°S, which is consistent with the proposed modifications on these habitats. It is worth noticing that mothers’ stronger reduction in *p*_*t*_ and *σ*_*t*_ due to ICE08 and CURL (Table 3) resulted in large differences in what can be considered ARS between groups (Fig. 2). By plotting expected *p* and *σ* values by quartiles (Fig. 4), maps indicated that males + tended to be overall more exploratory than mothers (*i.e.,* they reduced less *p* and *σ*). This could be attributed to different strategies elicited by distinct groups or just to the disproportional influence of some individuals, like the one reaching Maud Rise. To confirm this possible pattern more telemetry data is required south of 50°S.

As a synthesis we propose that humpback whales use the SS and surrounding waters as a high nutrient, high chlorophyll ecosystem where both high primary and secondary production are found^18,65,66^. The eastward flow of ACC and the interaction of these water masses with complex topography at SS allow their fertilization by Weddell sea and shelf-derived nutrients^65,66,73^. This expands the fertilization process to the east, downstream of ACC, promoting large phytoplankton blooms^71^ and large krill biomasses^72^. In addition, stratification provided by eddies and wind conditions favorable for Ekman pumping enhances photosynthesis by upwelling nutrient-rich waters and/or retaining phytoplankton in the upper mixed layer^90^. Finally, the marginal ice zone present at these areas provides shelter and food supply for krill during winter and an important source of nutrient supply for phytoplankton blooms during melting season^69,75,75–79^. The aforementioned conditions result in defining the dynamic feeding grounds for SWA humpback whales, which might be experimenting drastic modifications during the last years. It worth consideration though, that recent photo-identified SWA humpback whales at Western Antarctic Peninsula^61^ suggests that population recovery^34^ might promote that some individuals start using new and/or reoccupy historical areas. Continued, long-term monitoring of movements and habitat use of this population will help refining the results of this study and understanding how whales will respond to ongoing habitat modifications and population expanse.

## Conclusions

Summering grounds for humpback whales off SWA expands across a large area over the Southern Ocean, including historically recognized feeding grounds in the Scotia Sea, as well as adjacent areas over the North Weddell Ridge to the east, and the Weddell and Lazarev Seas. Oceanographic and topographic variables correlated with movement parameters agree with environmental features associated with large krill biomass occurrence. Inter-individual variation in movement patterns so far appears to be influenced by sex, with females accompanied by calves presenting patterns that are more consistent as a group than males^+^. Tracking duration south of 50°S represents the most important limitation for improving our understanding of humpback whales’ movement patterns, if their response to large habitat modification is to be thoroughly inspected in future. Joint estimation of movement parameters across individuals provides an efficient analytical approach for borrowing statistical strength in some whale groups (*e.g*., mothers), however, other sources of variation (*e.g*., age, social role, perhaps specifically for males) could be easily accommodated in the future as more data become available.

## Supplementary Information

Supplementary Information 1.

Supplementary Information 2.

## Data Availability

C++/TMB code for fitting the models, raw telemetry data and accompanying covariate data are available as Supplementary Information.
