# Supplementary material for: Individual and joint estimation of humpback whale migratory patterns and their environmental drivers in the Southwest Atlantic Ocean
Source: Sci Rep. 2022 May 6;12:7487. doi: 10.1038/s41598-022-11536-7 (PMC9076679; doi:10.1038/s41598-022-11536-7)
Supplement: Supplementary file 2 — Supplementary Information 2. [file 41598_2022_11536_MOESM2_ESM.docx]

**Supplementary information**

**Individual and joint estimation of humpback whale migratory patterns and their environmental drivers in the Southwest Atlantic Ocean**

*Luis Bedriñana-Romano^1,2,3^, Alexandre N. Zerbini^4,5,6^, Artur Andriolo^6,7^, Daniel Danilewicz^6, 8^ and Federico Sucunza^6, 8^

^1^Instituto de Ciencias Marinas y Limnológicas, Facultad de Ciencias, Universidad Austral de Chile, Casilla 567, Valdivia, Chile

^2^ NGO Centro Ballena Azul, Valdivia, Chile

^3^Centro de Investigación Oceanográfica COPAS Coastal, Universidad de Concepción, 4070043, Concepción, Región del Bio Bio, Chile

^4^Cooperative Institute for Climate, Ocean and Ecosystem Studies, University of Washington & Marine Mammal Laboratory Alaska Fisheries Science Center/NOAA 7600 Sand Point Way NE, Seattle, WA, USA

^5^Marine Ecology and Telemetry Research, 2468 Camp McKenzie Tr NW, Seabeck, WA, 98380, USA

^6^Instituto Aqualie, Av. Dr. Paulo Japiassú Coelho, 714, Sala 206, 36033-310, Juiz de Fora, MG, Brazil.

^7^Laboratório de Ecologia Comportamental e Bioacústica - LABEC, Departamento de Zoologia, Instituto de Ciências Biológicas, Universidade Federal de Juiz de Fora, Juiz de Fora, Minas Gerais, Brazil

^8^Grupo de Estudos de Mamíferos Aquáticos do Rio Grande do Sul (GEMARS), Porto Alegre, RS, Brazil

*Corresponding author: [luis.bedrinana.romano@gmail.com](mailto:luis.bedrinana.romano@gmail.com)

Table S1. Results for I-models. Maximum likelihood estimate for parameter coefficients and the corresponding standard error (SE) are provided. Significant parameter estimates (p≤0.05) are denoted in bold. Values for **ε_1_** and **ε_2_** were fixed at 0.001. The first four digits on the identification code (ID) correspond to tagging year. The sex column denotes whether whales were female (F), male (M) or undetermined (U). CP indicates calf presence. SR denotes the social role of each whale, with four possible categories, mother (Mo), adult (Ad), escort (Es) and undetermined (Un).

| **ID** | **Sex** | **CP** | **SR** | **A0** | **A0SE** | **A1** | **A1SE** | **A2** | **A2SE** | **A3** | **A3SE** | **A4** | **A4SE** | **A5** | **A5SE** |
| --- | --- | --- | --- | --- | --- | --- | --- | --- | --- | --- | --- | --- | --- | --- | --- |
| 2003-24642 | F | Yes | Mo | **8.61** | **0.06** | **-0.19** | **0.04** | **0.42** | **0.06** | **0.53** | **0.08** | 0.08 | 0.05 | **0.34** | **0.15** |
| 2005-10946 | F | Yes | Mo | **9.40** | **0.20** | -0.29 | 0.16 | 0.12 | 0.19 | 0.32 | 0.17 | 0.12 | 0.18 | **-83.14** | **32.34** |
| 2005-24641 | F | Yes | Mo | **9.11** | **0.25** | **-0.18** | **0.09** | 0.17 | 0.13 | 0.25 | 0.31 | 0.10 | 0.12 | 161.01 | 208.12 |
| 2009-87783 | F | Yes | Mo | **9.27** | **0.11** | **-0.47** | **0.12** | **0.75** | **0.08** | **-0.07** | **0.04** | **-0.58** | **0.09** | **-4.23** | **0.71** |
| 2009-87771 | U | No | Ad | **9.82** | **0.18** | 0.10 | 0.18 | 0.33 | 0.17 | **0.27** | **0.09** | -0.10 | 0.09 | **-3.76** | **0.82** |
| 2012-111871 | F | Yes | Mo | **8.63** | **0.13** | -0.01 | 0.06 | **1.16** | **0.19** | **0.07** | **0.02** | -0.07 | 0.04 | **0.35** | **0.10** |
| 2012-121189 | M | No | Ad | **9.14** | **0.04** | **-0.17** | **0.04** | -0.05 | 0.03 | 0.03 | 0.04 | **0.08** | **0.03** | **-0.75** | **0.09** |
| 2012-87632 | F | Yes | Mo | **9.10** | **0.79** | **-0.29** | **0.05** | 2.08 | 1.14 | -0.04 | 0.02 | -0.26 | 0.12 | 0.28 | 0.20 |
| 2017-172000 | U | No | Ad | **9.13** | **0.15** | **0.38** | **0.10** | 0.06 | 0.15 | **0.47** | **0.12** | **0.36** | **0.08** |  |  |
| 2017-172002 | F | Yes | Mo | **9.47** | **0.12** | **-0.43** | **0.05** | -0.01 | 0.07 | **0.25** | **0.06** | -0.06 | 0.06 | **-0.30** | **0.13** |
| 2017-84484 | M | No | Es | **8.63** | **0.11** | **-0.31** | **0.13** | **0.57** | **0.12** | 0.13 | 0.12 | 0.00 | 0.08 |  |  |
| 2017-111870 | M | No | Un | **9.09** | **0.12** | 0.01 | 0.02 | **1.45** | **0.18** | **0.12** | **0.05** | **-0.21** | **0.05** |  |  |
| 2017-172001 | F | Yes | Mo | **9.19** | **0.07** | **-0.42** | **0.06** | **0.53** | **0.09** | 0.00 | 0.04 | **-0.15** | **0.04** | **-0.92** | **0.11** |
| 2017-121203 | M | No | Es | **8.98** | **0.08** | **0.13** | **0.04** | **0.99** | **0.14** | **-0.20** | **0.05** | **-0.28** | **0.04** | **0.29** | **0.09** |
| 2017-120937 | M | No | Es | **8.97** | **0.10** | 0.02 | 0.07 | **1.50** | **0.21** | **-0.12** | **0.06** | 0.02 | 0.04 |  |  |
| 2018-84485 | F | Yes | Mo | **8.63** | **0.14** | **-0.44** | **0.05** | **0.83** | **0.08** | -0.15 | 0.08 | **-0.18** | **0.06** |  |  |
| 2018-112696 | F | Yes | Mo | **9.76** | **0.22** | **-0.47** | **0.17** | **0.39** | **0.17** | -0.14 | 0.24 | 0.07 | 0.10 |  |  |
| 2018-172008 | M | No | Es | **9.58** | **0.09** | 0.08 | 0.05 | -0.10 | 0.07 | 0.03 | 0.04 | **-0.32** | **0.04** | **-1.38** | **0.14** |
| 2018-121191 | F | Yes | Mo | **9.69** | **0.29** | **-0.24** | **0.08** | 0.11 | 0.10 | **-0.62** | **0.16** | **0.31** | **0.09** |  |  |
| 2018-171994 | M | No | Es | **8.91** | **0.08** | **-0.15** | **0.07** | **0.48** | **0.06** | 0.10 | 0.08 | -0.01 | 0.07 |  |  |
| 2019-194591 | U | No | Ad | **8.59** | **0.06** | **-0.36** | **0.05** | **0.53** | **0.07** | 0.07 | 0.11 | -0.01 | 0.04 |  |  |
| 2019-194601 | F | Yes | Mo | **8.19** | **0.09** | **-0.37** | **0.10** | **0.73** | **0.11** | **1.28** | **0.29** | **0.84** | **0.18** |  |  |
|  |  |  |  |  |  |  |  |  |  |  |  |  |  |  |  |
| **ID** | **Sex** | **CP** | **SR** | **B0** | **B0SE** | **B1** | **B1SE** | **B2** | **B2SE** | **B3** | **B3SE** | **B4** | **B4SE** | **B5** | **B5SE** |
| 2003-24642 | F | Yes | Mo | **0.85** | **0.25** | **-1.78** | **0.26** | 0.19 | 0.16 | **-5.22** | **0.47** | **0.63** | **0.16** | **7.49** | **1.14** |
| 2005-10946 | F | Yes | Mo | **-2.27** | **0.37** | 0.42 | 0.34 | 0.46 | 0.39 | -0.48 | 0.32 | -0.48 | 0.36 | **285.89** | **101.00** |
| 2005-24641 | F | Yes | Mo | **-2.23** | **0.52** | **0.73** | **0.27** | 0.45 | 0.39 | 1.03 | 0.56 | 0.47 | 0.28 | 369.31 | 316.39 |
| 2009-87783 | F | Yes | Mo | **-1.32** | **0.18** | **0.71** | **0.30** | **-1.73** | **0.12** | 0.15 | 0.08 | **1.51** | **0.16** | **3.95** | **1.58** |
| 2009-87771 | U | No | Ad | **-2.53** | **0.24** | 0.20 | 0.26 | **-0.56** | **0.21** | -0.09 | 0.22 | **0.47** | **0.13** | **8.27** | **1.73** |
| 2012-111871 | F | Yes | Mo | **0.97** | **0.27** | 0.28 | 0.14 | **-2.05** | **0.24** | **-0.43** | **0.11** | **0.40** | **0.12** | **12.63** | **2.87** |
| 2012-121189 | M | No | Ad | 0.12 | 0.19 | **1.14** | **0.27** | **-0.29** | **0.13** | 0.01 | 0.16 | -0.04 | 0.11 | 0.24 | 0.52 |
| 2012-87632 | F | Yes | Mo | **78.53** | **13.55** | **4.21** | **1.56** | **93.06** | **19.14** | **4.41** | **0.64** | **-10.07** | **1.85** | **10.59** | **4.82** |
| 2017-172000 | U | No | Ad | **-1.25** | **0.25** | **-0.47** | **0.20** | 0.19 | 0.23 | **-0.83** | **0.19** | **-0.76** | **0.13** |  |  |
| 2017-172002 | F | Yes | Mo | **-1.97** | **0.17** | **0.78** | **0.07** | **0.48** | **0.11** | **-1.10** | **0.14** | 0.15 | 0.12 | **2.37** | **0.39** |
| 2017-84484 | M | No | Es | -0.09 | 0.29 | **0.68** | **0.29** | **-1.22** | **0.23** | -0.35 | 0.26 | -0.05 | 0.13 |  |  |
| 2017-111870 | M | No | Un | **-0.80** | **0.17** | **0.12** | **0.06** | **-1.87** | **0.20** | **-0.48** | **0.13** | **0.44** | **0.08** |  |  |
| 2017-172001 | F | Yes | Mo | **-1.19** | **0.12** | **0.93** | **0.14** | **-0.64** | **0.12** | **-0.42** | **0.11** | **0.28** | **0.06** | **4.25** | **0.35** |
| 2017-121203 | M | No | Es | **-0.29** | **0.12** | -0.11 | 0.09 | **-1.54** | **0.18** | **0.29** | **0.12** | **0.55** | **0.08** | -0.21 | 0.32 |
| 2017-120937 | M | No | Es | **-0.88** | **0.14** | **-0.41** | **0.17** | **-1.88** | **0.26** | -0.10 | 0.12 | **-0.20** | **0.10** |  |  |
| 2018-84485 | F | Yes | Mo | **1.06** | **0.32** | **0.76** | **0.16** | **-1.79** | **0.17** | -0.09 | 0.16 | **0.51** | **0.13** |  |  |
| 2018-112696 | F | Yes | Mo | **-2.58** | **0.30** | **0.68** | **0.27** | **-0.54** | **0.20** | 0.44 | 0.32 | 0.00 | 0.12 |  |  |
| 2018-172008 | M | No | Es | **-1.17** | **0.14** | **-0.32** | **0.11** | -0.19 | 0.13 | **0.31** | **0.09** | **0.63** | **0.09** | **1.63** | **0.27** |
| 2018-121191 | F | Yes | Mo | **-2.31** | **0.38** | **0.43** | **0.16** | -0.14 | 0.16 | **1.27** | **0.25** | **-0.40** | **0.14** |  |  |
| 2018-171994 | M | No | Es | 0.18 | 0.21 | 0.09 | 0.13 | **-0.76** | **0.09** | **-0.78** | **0.20** | 0.15 | 0.13 |  |  |
| 2019-194591 | U | No | Ad | **-0.53** | **0.14** | **0.37** | **0.10** | **-0.50** | **0.15** | 0.15 | 0.23 | **-0.26** | **0.08** |  |  |
| 2019-194601 | F | Yes | Mo | **1.23** | **0.30** | **2.54** | **0.19** | **-1.60** | **0.23** | **-0.97** | **0.33** | **-0.88** | **0.23** |  |  |


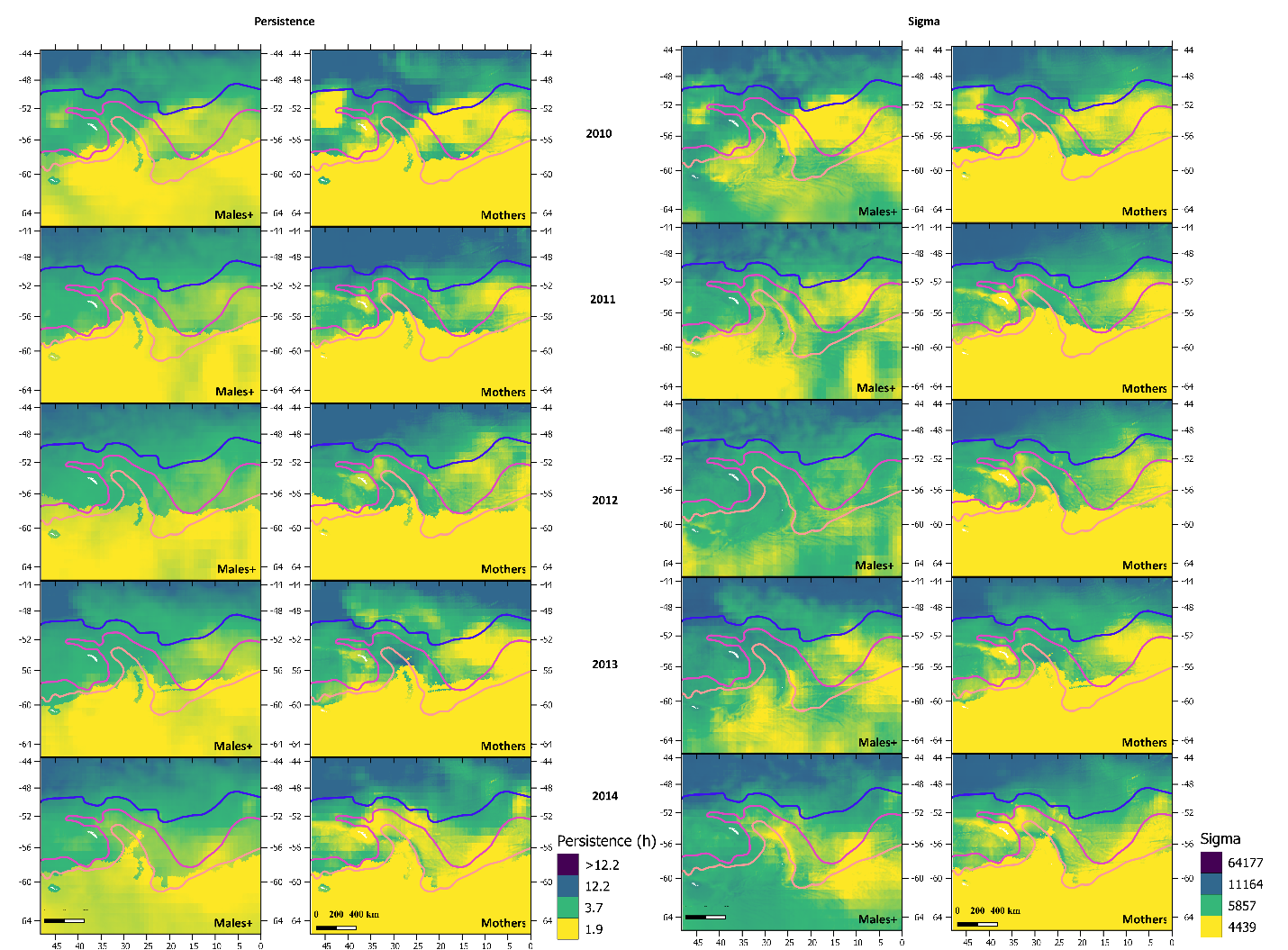


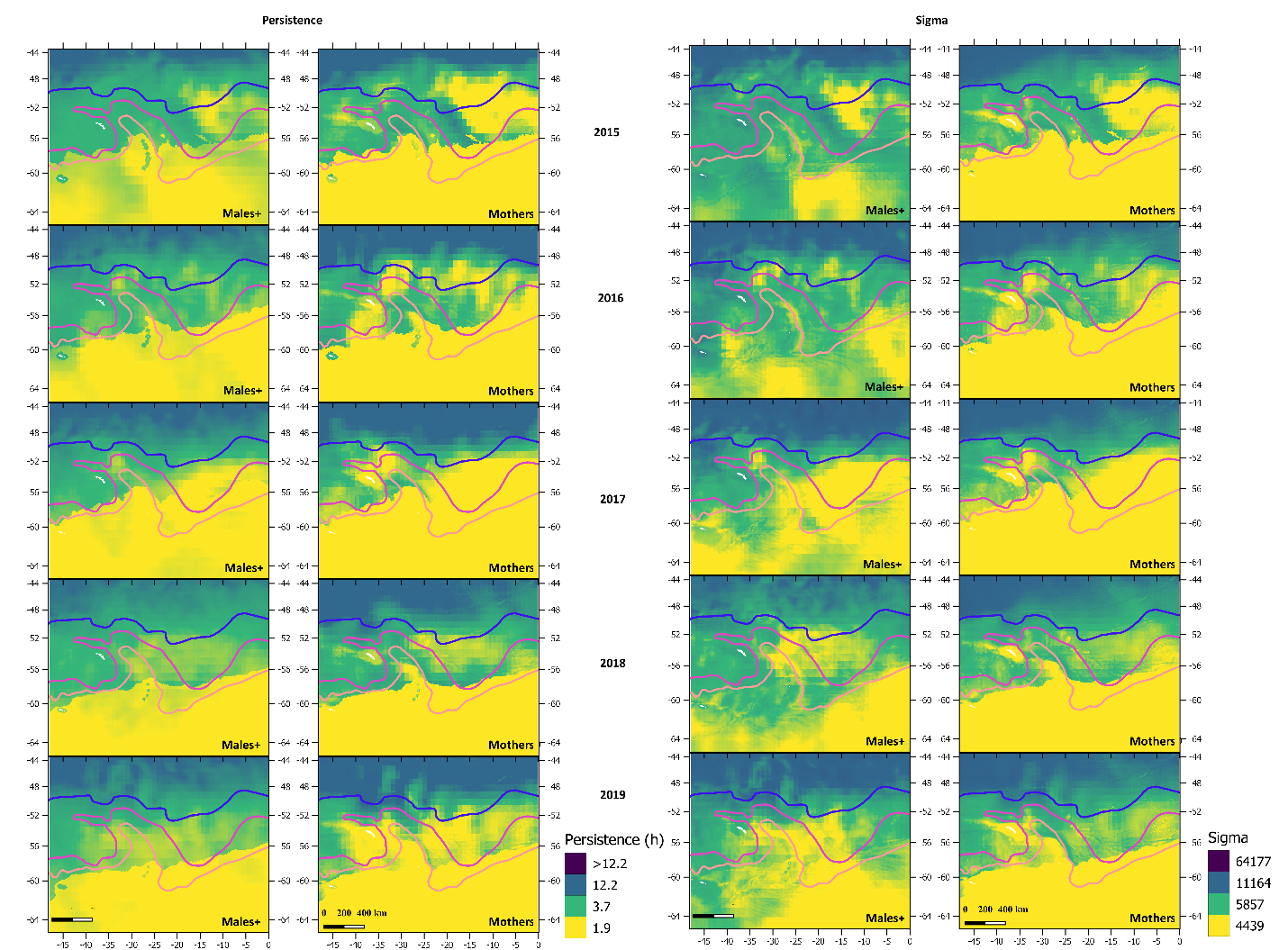


Figure S1. Predicted sigma (σ) and persistence (*p*=3/β) for males+ and mothers, considering environmental conditions of each January from 2010 to 2019, using the best P-model. From north to south, colored lines denote the location of polar front (PF, blue), Southern Antarctic Circumpolar Current Front (SACCF, fuchsia), and the southern boundary of the Antarctic circumpolar current (SBACCF, pink). Antarctic circumpolar fronts data is made publicly available by the Australian Antarctic Data Centre (<https://researchdata.edu.au>). Data layers were created in R ver. 4.0.2 ([www.r-project.org](http://www.r-project.org)) and ensembled in QGIS ver. 3.8.0 ([www.qgis.org](http://www.qgis.org)) for final rendering.


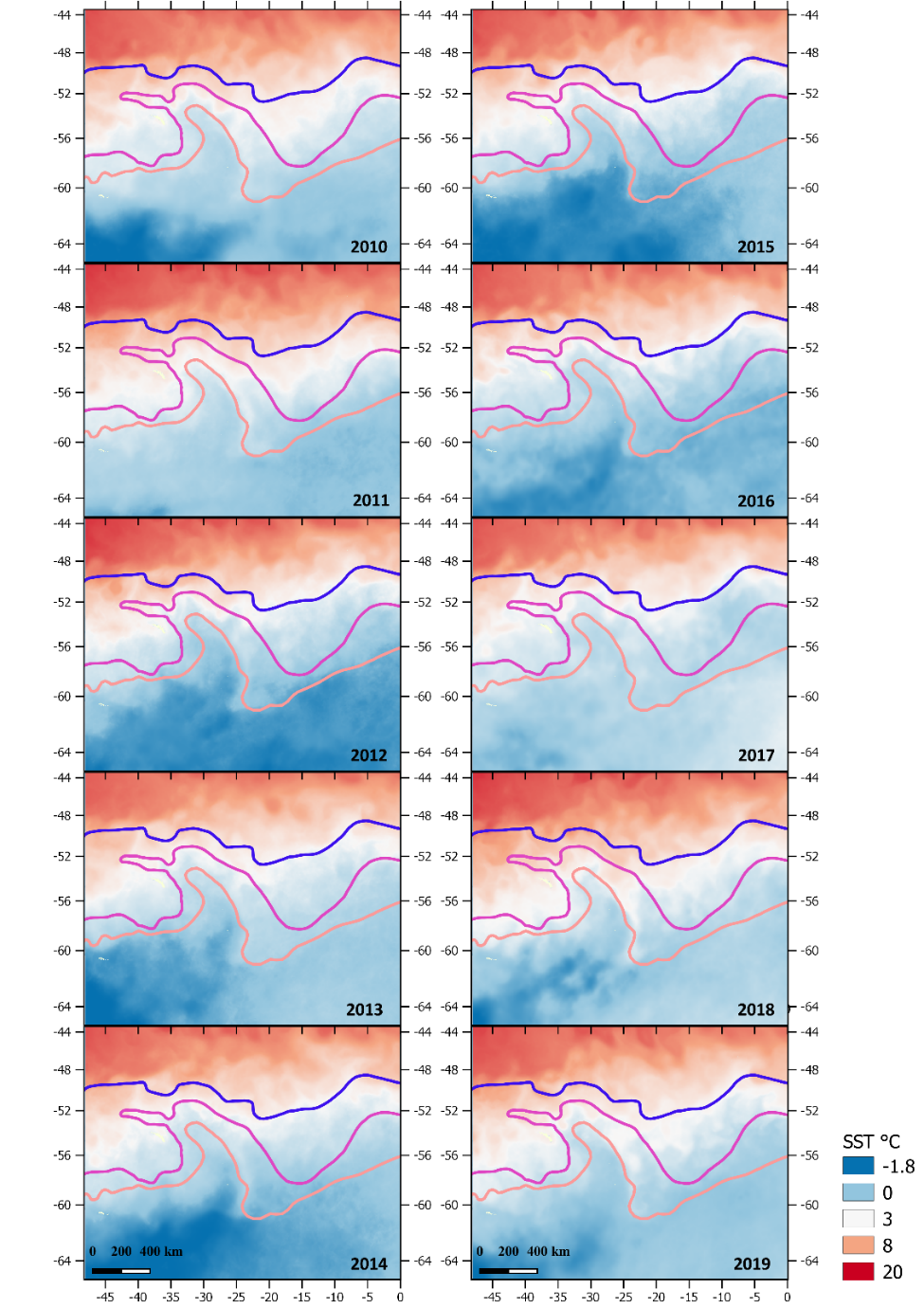


Figure S2. Monthly average SST for January from 2010 to 2019. From north to south, colored lines denote the location of polar front (PF), Southern Antarctic Circumpolar Current Front (SACCF), and the southern boundary of the Antarctic circumpolar current (SBACCF). Antarctic circumpolar fronts data is made publicly available by the Australian Antarctic Data Centre (<https://researchdata.edu.au>). Data layers were created in R ver. 4.0.2 ([www.r-project.org](http://www.r-project.org)) and ensembled in QGIS ver. 3.8.0 ([www.qgis.org](http://www.qgis.org)) for final rendering.


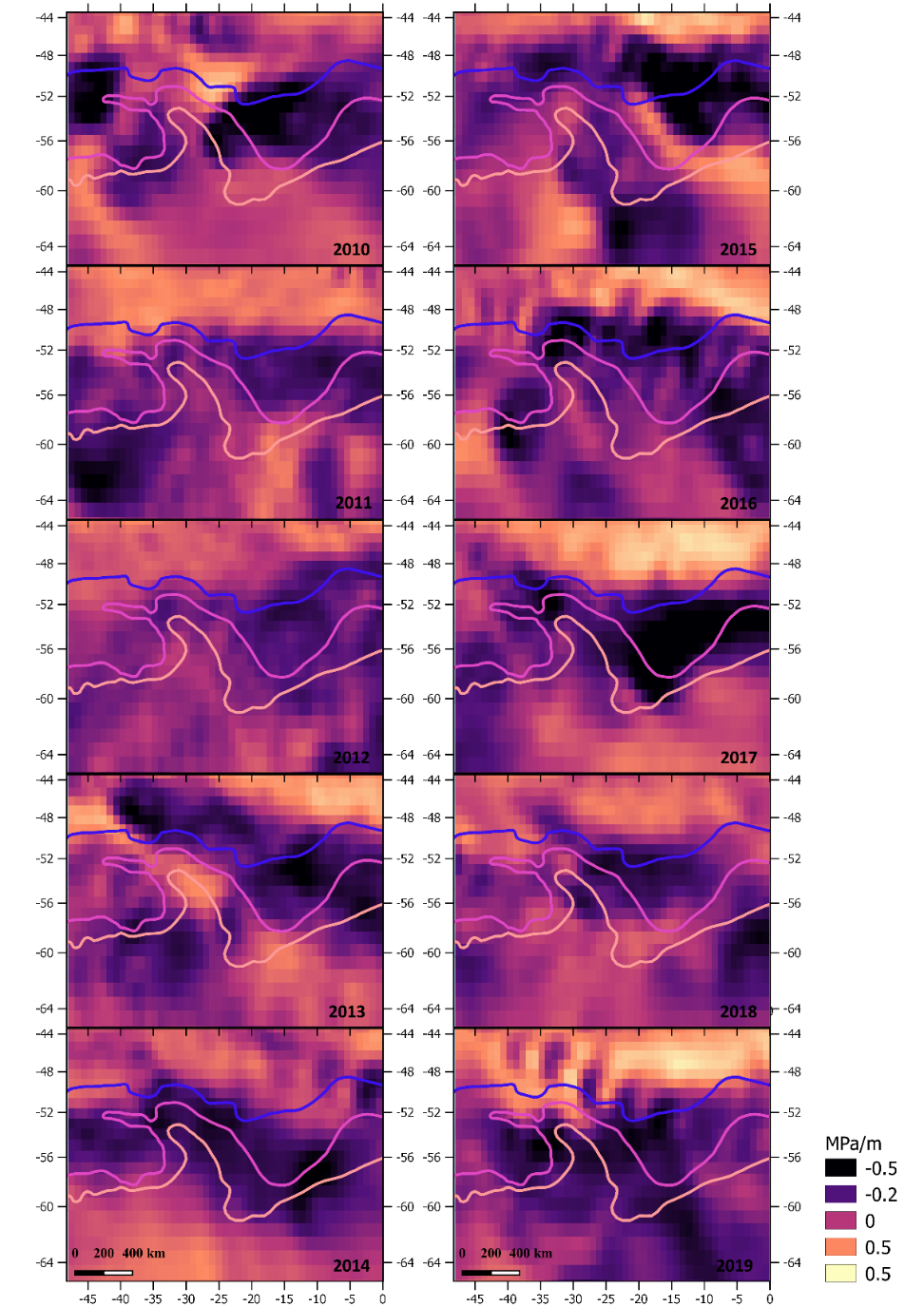


Figure S3. Monthly average CURL for January from 2010 to 2019. From north to south, colored lines denote the location of polar front (PF), Southern Antarctic Circumpolar Current Front (SACCF), and the southern boundary of the Antarctic circumpolar current (SBACCF). Antarctic circumpolar fronts data is made publicly available by the Australian Antarctic Data Centre (<https://researchdata.edu.au>). Data layers were created in R ver. 4.0.2 ([www.r-project.org](http://www.r-project.org)) and ensembled in QGIS ver. 3.8.0 ([www.qgis.org](http://www.qgis.org)) for final rendering.


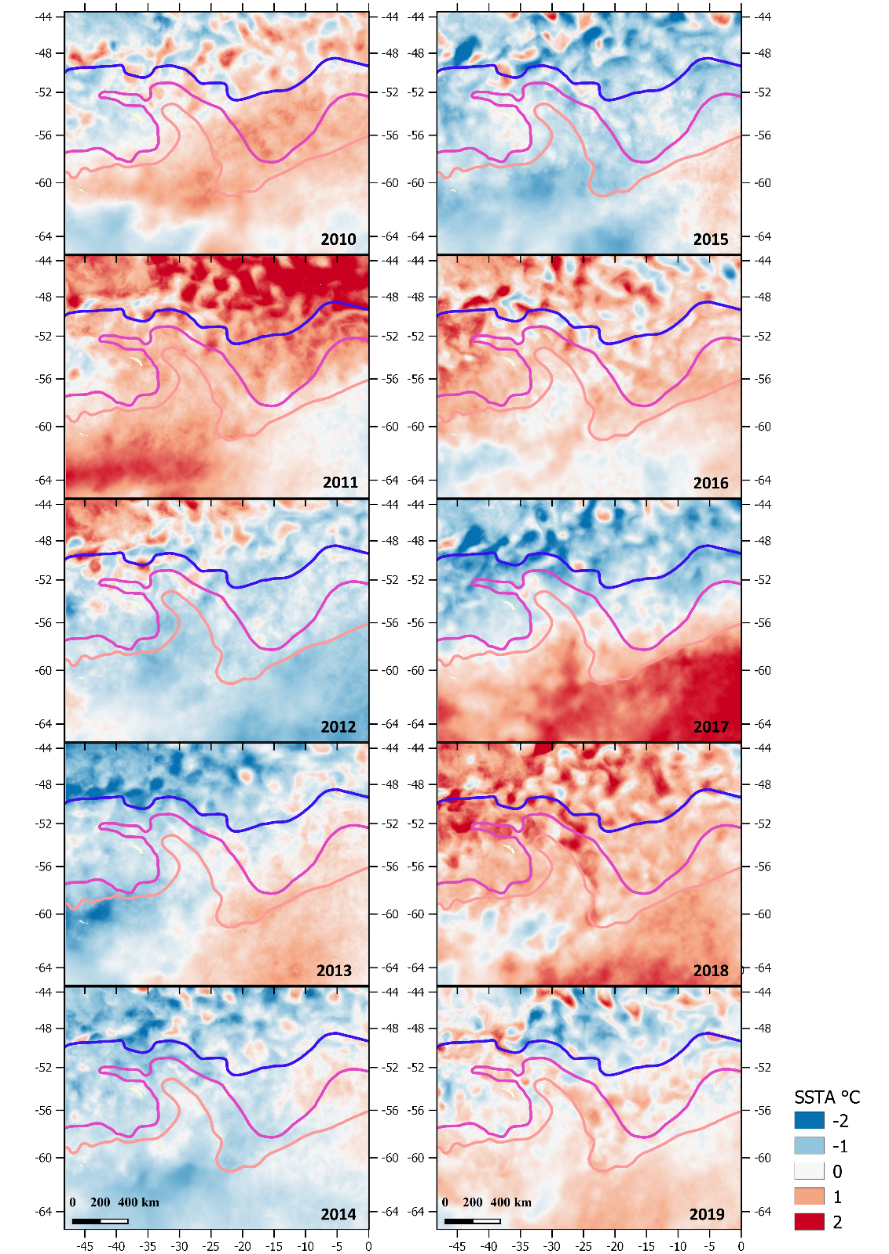


Figure S4. Monthly average SSTA for January from 2010 to 2019. From north to south, colored lines denote the location of polar front (PF), Southern Antarctic Circumpolar Current Front (SACCF), and the southern boundary of the Antarctic circumpolar current (SBACCF). Antarctic circumpolar fronts data is made publicly available by the Australian Antarctic Data Centre (<https://researchdata.edu.au>). Data layers were created in R ver. 4.0.2 ([www.r-project.org](http://www.r-project.org)) and ensembled in QGIS ver. 3.8.0 ([www.qgis.org](http://www.qgis.org)) for final rendering.

**Movement model**

TMB/C++ code for fitting the population model (P-model) described in the main text. I-models can be found in supplementary files from Bedriñana-Romano, L., Hucke-Gaete, R., Viddi, F.A., Johnson, D., Zerbini, A.N., Morales, J., Mate, B., Palacios, D.M., 2021. Defining priority areas for blue whale conservation and investigating overlap with vessel traffic in Chilean Patagonia, using a fast-fitting movement model. Scientific Reports 11, 2709. <https://doi.org/10.1038/s41598-021-82220-5>

#include <TMB.hpp>

template<class Type>

Type objective_function<Type>::operator() ()

{

// Data//

DATA_INTEGER(ind); // number of animals

DATA_VECTOR(x);

DATA_VECTOR(y);

DATA_FACTOR(id);

DATA_VECTOR(delta);

DATA_VECTOR(errx);

DATA_VECTOR(erry);

DATA_SCALAR(sd1);

DATA_SCALAR(sd2);

DATA_MATRIX(Ialpha1);

DATA_MATRIX(Ialpha2);

DATA_SCALAR(sd_beta);

DATA_SCALAR(sd_sigma);

DATA_VECTOR(prof);

DATA_VECTOR(sst);

DATA_VECTOR(ssta);

//DATA_VECTOR(chl);

DATA_VECTOR(curl);

DATA_VECTOR(ice08);

DATA_VECTOR(G);

//Parameters//

PARAMETER_MATRIX(alpha1); // rbind(u1,V1), i.e., 2xN matrix where alpha1[,1] = c(u1[1], V1[1])

PARAMETER_MATRIX(alpha2); // rbind(u2,V2)

PARAMETER_VECTOR(log_sigma);

PARAMETER_VECTOR(log_beta);

PARAMETER(B0);

PARAMETER(A0);

PARAMETER(A1);

PARAMETER(A2);

PARAMETER(A3);

PARAMETER(A4);

PARAMETER(A5);

PARAMETER(B1);

PARAMETER(B2);

PARAMETER(B3);

PARAMETER(B4);

PARAMETER(B5);

PARAMETER(a0);

PARAMETER(a1);

PARAMETER(a2);

PARAMETER(a3);

PARAMETER(a4);

PARAMETER(a5);

PARAMETER(b0);

PARAMETER(b1);

PARAMETER(b2);

PARAMETER(b3);

PARAMETER(b4);

PARAMETER(b5);

//PARAMETER(log_sd_sigma);

//PARAMETER(log_sd_beta);

//Transform Parameters//

//Type sd_sigma = exp(log_sd_sigma);

//Type sd_beta = exp(log_sd_beta);

int i,j;

matrix<Type> mean_alpha1(2,1);

matrix<Type> mean_alpha2(2,1);

Type nll = Type(0);

// define bivariate state vectors, matrices, etc...

matrix<Type> T(2,2);

matrix<Type> Q(2,2);

vector<Type> sigma=exp(log_sigma);

vector<Type> beta=exp(log_beta);

//Process model

for(j = 0; j < ind; ++j) {

//Initial conditions

T.fill(0); T(0,0) = 1.0;

Q.fill(0);

Q(0,0) = sd1*sd1;

Q(1,1) = sd2*sd2;

mean_alpha1 = T * Ialpha1.col(j);

mean_alpha2 = T * Ialpha2.col(j);

nll += density::MVNORM(Q)(alpha1.col(id(j))-mean_alpha1); //

nll += density::MVNORM(Q)(alpha2.col(id(j))-mean_alpha2); // MVNORM_t returns -log mvn density

nll -= dnorm(log_beta(id(j)), (B0+b0*G(id(j))) + (B1+b1*G(id(j))) *prof(id(j))+ (B2+b2*G(id(j)))*sst(id(j))+ (B3+b3*G(id(j)))*curl(id(j))+ (B4+b4*G(id(j)))*ssta(id(j)) + (B5+b5*G(id(j)))*ice08(id(j)), sd_beta, true);

nll -= dnorm(log_sigma(id(j)), (A0+a0*G(id(j))) + (A1+a1*G(id(j))) *prof(id(j))+ (A2+a2*G(id(j)))*sst(id(j))+ (A3+a3*G(id(j)))*curl(id(j))+ (A4+a4*G(id(j)))*ssta(id(j)) + (A5+a5*G(id(j)))*ice08(id(j)), sd_sigma, true);

for(i = (id(j)+1); i < id(j+1); ++i){

nll -= dnorm(log_beta(i), (B0+b0*G(i)) + (B1+b1*G(i)) *prof(i)+ (B2+b2*G(i))*sst(i)+ (B3+b3*G(i))*curl(i)+ (B4+b4*G(i))*ssta(i)+ (B5+b5*G(i))*ice08(i), sd_beta, true);

nll -= dnorm(log_sigma(i), (A0+a0*G(i)) + (A1+a1*G(i)) *prof(i)+ (A2+a2*G(i))*sst(i)+ (A3+a3*G(i))*curl(i)+ (A4+a4*G(i))*ssta(i)+ (A5+a5*G(i))*ice08(i), sd_sigma, true);

T(0,1) = (1-exp(-beta(i)*delta(i-1)))/beta(i);

T(1,1) = exp(-beta(i)*delta(i-1));

Q(0,0) = (sigma(i)*sigma(i))*(delta(i-1)-(2/beta(i))*(1-exp(-beta(i)*delta(i-1)))+(1/(2*beta(i)))*(1-exp(-2*beta(i)*delta(i-1))));

Q(0,1) = 0.5*(sigma(i)*sigma(i))*(1 - 2*exp(-beta(i)*delta(i-1)) + exp(-2*beta(i)*delta(i-1)));

Q(1,0) = Q(0,1);

Q(1,1) = 0.5*(sigma(i)*sigma(i))*beta(i)*(1-exp(-2*beta(i)*delta(i-1)));

mean_alpha1 = T * alpha1.col(i-1);

mean_alpha2 = T * alpha2.col(i-1);

nll += density::MVNORM(Q)(alpha1.col(i)-mean_alpha1);

nll += density::MVNORM(Q)(alpha2.col(i)-mean_alpha2);

}

}

//Observation model

for( int i=0;i < x.size();i++) {

nll-=dnorm(x(i),alpha1(0,i),errx(i),true);

nll-=dnorm(y(i),alpha2(0,i),erry(i),true);

}

ADREPORT(alpha1);

ADREPORT(alpha2);

ADREPORT(log_sigma);

ADREPORT(log_beta);

ADREPORT(B0);

ADREPORT(A0);

ADREPORT(A1);

ADREPORT(A2);

ADREPORT(A3);

ADREPORT(A4);

ADREPORT(A5);

ADREPORT(B1);

ADREPORT(B2);

ADREPORT(B3);

ADREPORT(B4);

ADREPORT(B5);

ADREPORT(a0);

ADREPORT(a1);

ADREPORT(a2);

ADREPORT(a3);

ADREPORT(a4);

ADREPORT(a5);

ADREPORT(b0);

ADREPORT(b1);

ADREPORT(b2);

ADREPORT(b3);

ADREPORT(b4);

ADREPORT(b5);

//ADREPORT(sd_sigma);

//ADREPORT(sd_beta);

return nll;

}
